# The histone acetylation-related gene signature predicts prognosis and immunotherapy response in stomach adenocarcinoma

**DOI:** 10.3389/fonc.2025.1527253

**Published:** 2025-09-02

**Authors:** Chen Dai, Rishun Su, Zhenzhen Zhao, Yangyang Guo, Wenbin Hou, Yulong He, Guangyao Liu, Songcheng Yin, Changhua Zhang

**Affiliations:** ^1^ Digestive Diseases Center, The Seventh Affiliated Hospital of Sun Yat-sen University, Shenzhen, Guangdong, China; ^2^ Guangdong Provincial Key Laboratory of Digestive Cancer Research, The Seventh Affiliated Hospital of Sun Yat-sen University, Shenzhen, Guangdong, China; ^3^ Key Laboratory of Diagnosis and Treatment of Severe Hepato-Pancreatic Diseases of Zhejiang Province, The First Affiliated Hospital of Wenzhou Medical University, Wenzhou, Zhejiang, China; ^4^ Department of Gastrointestinal Surgery, The First Affiliated Hospital of Sun Yat-Sen University, Shenzhen, Guangzhou, China; ^5^ Department of Gastrointestinal Surgery, Affiliated Yijishan Hospital, Wannan Medical College, Wuhu, Anhui, China

**Keywords:** stomach adenocarcinoma, histone acetylation, prognostic signature, immunotherapy, DCLK1

## Abstract

**Background:**

Gastric cancer (GC) is a very aggressive, with extreme heterogeneity and rapid growth, most frequently manifested histologically as stomach adenocarcinoma (STAD). Current evidence suggests that histone acetylation is critical for the origin and development of tumors. However, the significance of histone acetylation-related gene signatures for prognosis of STAD patients and mechanisms of histone acetylation in STAD therapy remains unclear.

**Methods:**

We identified histone acetylation-related genes in STAD from TCGA and constructed eight-gene signatures by utilizing a univariate Cox regression model with the Least Absolute Shrinkage and Selection Operator (LASSO). In addition, a nomogram was plotted to predict the prognostic significance of the established risk model. We examined associations between our gene signature and somatic mutation, immune subtype, clinicopathological features, tumor microenvironment, immune cell infiltration and immune activity, immunotherapy prediction and drug sensitivity. Cell-based assays were performed to determine the relationship between Doublecortin Like Kinase 1 (DCLK1) and the proliferation, migration and oxaliplatin resistance of GC cells *in vitro*. Immunohistochemical experiments were used to explore the relationship between DCLK1 and clinicopathological parameters.

**Results:**

A prognostic model composed of eight histone acetylation-related genes in STAD was developed. Based on median risk score, the STAD patients were equally assigned into two groups of high- and low-risk, where high-risk represented a less favorable prognosis than low-risk. The two groups showed significant differences with respect to somatic mutation, immune subtype, clinicopathological features, tumor microenvironment, immune cell infiltration and immune activity, immunotherapy prediction and drug sensitivity. The results generated during Gene Ontology (GO) and Kyoto Encyclopedia of Genes and Genomes (KEGG) analyses suggested that Differentially Expressed Genes (DEGs) in the two groups were involved in cancer-related processes and pathways. Cell-based assays indicated that DCLK1 is a promoting factor in gastric cancer and can promote oxaliplatin resistance in gastric cancer cells. Increased DCLK1 expression was also associated with Borrmann’s Type, depth of invasion, lymph node metastasis, and TNM stage in GC patients.

**Conclusion:**

A novel histone acetylation-related gene signature, which possesses potential value in predicting the prognosis and immunotherapy effectiveness regarding STAD patients, was developed. This signature may serve as a reliable biomarker for prognosis of STAD and promote the identification of novel treatment targets for STAD. Furthermore, DCLK1 exhibited oncogenic roles and may be a new target for STAD therapy.

## 1 Introduction

Gastric cancer (GC) is a very aggressive cancer, with extreme heterogeneity and rapid growth, and high incidence rate and mortality rates (1, 2). Annual diagnoses are over 1 million worldwide, with annual fatalities reaching 76,000 based on the Global Cancer Report 2020 (1). The most frequent histological type of GC is stomach adenocarcinoma (STAD). Although substantial clinical advances including radical surgery, chemotherapy, radiotherapy, molecularly targeted drugs have been applied in early therapy of patients with GC over the past several decades, diagnostic efficiency and therapeutic effects of advanced GC remain unsatisfactory and the five-year survival rate of advanced GC has been slow to improve (3–5). Therefore, reliable novel biomarkers and prognostic models that can be used to improve prognostic predictions and reasonable therapeutic strategies are urgently required for patients with STAD.

Epigenetic alterations, including DNA methylation, histone modifications and chromatin remodeling refer to heritable phenotype changes in gene expression with the DNA sequence unchanged (6, 7). As one of epigenetic alterations, histone modifications vary, including methylation, acetylation, ubiquitination and phosphorylation (8). Acetylation modification of histone is a reversible dynamic process that achieves a balance between histone acetyltransferases (HATs) and histone deacetylases (HDACs) (9, 10). HATs catalyze the transfer of acetyl from acetyl coenzyme-A to N-terminal lysine in histone, which facilitates the binding of transcription factors to promoters and promotes the transcription of genes (11–13). In contrast, HDAC catalyze hydrolysis of acetyl from lysine, generating opposite results of HAT (14). Current research indicates that histone acetylation are important for tumor origin and progression (15, 16). For example, Wisnieski et al. showed that acetylation levels increased in some regions of CDKN1A and these modifications were significantly associated with gastric cancer (17). Tani et al. noted that histone H3 and H4 acetylation play an important role in the abnormal expression of MYO18B, contributing to the origin and progression of lung cancer (18). Falahi et al. showed that decrease of H4K16 (monoacetylated lysine 16 of histone H4) acetylation is closely associated with the early process of breast cancer (19). Other studies have shown that HAT/HDAC inhibitors, as a class of newly developing antitumor drugs, may alter the expression of oncogenes or tumor suppressor genes by changing histone acetylation levels. However, the role of histone acetylation-related gene signature in prognosis of patients with STAD and mechanisms of histone acetylation in STAD therapy remain unclear.

Therefore, we performed a systematic study to classify TCGA-STAD patients based on histone acetylation-related genes and screen prognostic significance of histone acetylation-related DEGs between two clusters. We also established histone acetylation-related gene signature to obtain the risk score for evaluating prognosis and conducted functional enrichment analysis to investigate the underlying mechanisms. The association between our gene signature and somatic mutation, tumor microenvironment, immune cell infiltration, immune activity and drug sensitivity were explored to determine novel strategies for targeted treatment of STAD. We selected the gene with the largest correlation coefficient in the prognostic model for subsequent experimental analysis and assessment.

## 2 Materials and methods

### 2.1 Data collection

Transcriptome sequencing data of STAD patients and matching clinical features were sourced from the TCGA database. Validation cohorts were acquired from GSE84437.

### 2.2 Tumor classification based on histone acetylation-related DEGs

We used the R package “ConsensusClusterPlus” to further investigate the associations between histone acetylation-related genes and STAD subtypes and determine number and stability of clusters. Based on the cumulative distribution functions, the optimal cluster number of k-value was obtained (20). The KM survival analysis was conducted using the R package “survival.” Differential genes between the two clusters and histone acetylation-related genes are shown in **Supplementary Tables 1, 2**.

### 2.3 Establishment and verification of prognostic histone acetylation-related gene signature

The univariate Cox regression algorithm was employed to filter out histone acetylation-related genes independently related to prognosis, and LASSO Cox regression was carried out to further narrow the range of candidate genes using the R package “glmnet.” The risk scores were calculated for STAD patients as $Risk\;Score=\sum_{i}^{n}Xi*Yi$ (X: coefficients, Y: gene expression level), and based on the middle value, the STAD patients were equally assigned into low- and high-risk groups. Then, Kaplan-Meier analysis was conducted, and PCA, t-SNE and ROC curves were plotted. The R package “pheatmap” was applied to draw the heatmap and applied the GSE84437 cohort to validate the risk signature. The prognostic significance of the signature was analyzed with univariate and multivariable Cox regression. The Eight-Gene Prognostic Model is shown in **Supplementary Table 3**.

### 2.4 Establishment of nomogram

A nomogram based on independent prognostic parameters was developed using R package “rms” and “regplot” for STAD patients to predict the survival probability. In addition, calibration curve and AUC were applied to evaluate the nomogram regarding accuracy, and decision curves analysis was also performed to confirm the model with respect to its clinical effectiveness.

### 2.5 Somatic mutation, tumor immunity, immunotherapy prediction and drug susceptibility analysis

The TCGA database was used to retrieve sample mutation data, and the R package “maftools” was used to estimate the mutation status between the two groups. Immune, Stromal and ESTIMATE scores were applied to evaluate association between the risk score and tumor microenvironment (TME) with “ESTIMATE” R package. Immune cell subtype infiltration values of TCGA-STAD dataset samples were based on seven algorithms: XCELL, TIMER, QUANTISEQ, MCPcounter, EPIC, CIBERSORT-ABS and CIBERSORT (21–30). The correlation between infiltration immune cell subtypes and risk scores was established through the application of Spearman correlation analysis with R packages “ggpubr.” In addition, different immune-checkpoint genes in our two risk groups were confirmed with respect to their expression by using R packages “limma.” MSI, TMB, TIDE scores and the response to CTLA4 inhibitor were performed to assess and predict immunotherapy effectiveness of our prognostic signature. IC50 (Half-maximal inhibitory concentration) was used to determine the relationship between our prognostic signature and various drug susceptibility.

### 2.6 Functional enrichment analysis

GO and KEGG analyses using “clusterProfiler” R package were performed to examine the mechanisms of the signature, and GSEA analysis was used to elucidate signaling pathways.

### 2.7 Cell lines and cell culture

The human immortalized normal gastric epithelial cell (GES1) and gastric adenocarcinoma cell lines (MKN-28 and HGC-27) were provided by the Chinese Academy of Sciences (Shanghai). These cells were maintained at 37°C and in 5% CO_2_ in RPMI-1640 (Gibco, USA) medium with 10% fetal bovine serum (Gibco, USA) and 1% penicillin-streptomycin. Over one year, Oxaliplatin-resistant GC cell line HGC-27R was established stepwise through constant exposure to increasing quantities of Oxaliplatin and culture in RPMI-1640 (Gibco, USA) medium with oxaliplatin (1 uM).

### 2.8 Cell transfection

The small interfering RNAs (siRNAs) targeting DCLK1 and scrambled siRNA nontarget control (Si-NC) were supplied by TRANSHEEPBIO (China), and their sequences are as follows: Si-DCLK1-1: sense UCUGUCGGAUAACGUGAAUUU and antisense AAAUUCACGUUAUCCGACAGA; Si-DCLK1-2: sense ACCCGAACUCUGUCGGAUAAC and antisense GUUAUCCGACAGAGUUCGGGU (**Supplementary Table 4**). Six-well plates were seeded with GC cell at a density of 2.0 × 10^5^/well and then Namipo™ was used to assist transfection in accordance with the instructions from manufacturer.

### 2.9 Isolation of total RNA with quantitative reverse transcription real-time polymerase chain reaction

Total RNA from cell lines was obtained with RNA Isolater Total RNA Extraction Reagent (Vazyme, Nanjing, China) in accordance with the instructions from manufacturer. Subsequently, cDNA was generated utilizing the Evo M-MLV RT Premix kit (Accurate Biotechnology). Then, qRT-PCR assay was performed using SYBR Green Master Mix (Vazyme), with beta-actin as the normalization control and the 2−ΔΔCt method for the quantification of RNA expression levels. The primer sequences (5′-3′) were as follows: DCLK1 forward, ACTTCGACGAGCGGGATAAG and reverse, GGGCCTCAAAAGATCGGAACC; β-Actin forward, CACCATTGGCAATGAGCGGTTC and reverse, AGGTCTTTGCGGATGTCCACGT (**Supplementary Table 4**).

### 2.10 Cell proliferation assay

Cell viability and proliferation were assayed by Cell Counting Kit-8 kit (MeilunBio, Guangzhou, China) following the instructions from manufacturer. On the first day, 2 × 10^3^ cells per well were seeded into 96-well plates for culture. Then on day 1, 2, 3, 4 and 5 follow cell seeding, the plates were added with 10 ul of CCK-8 reagent into each of wells and incubated for 1 h at 37°C to detect cell viability. The 450-nm optical absorption values were recorded using a microplate reader (Thermo, U.S.A.) on days 1, 2, 3, 4, and 5.

### 2.11 Cytotoxicity of oxaliplatin *in vitro*

Each well of a 96 well plate was seeded with 10,000 cells and treated with different concentrations of oxaliplatin (Aladdin, Shanghai) after 24 h. The Cell Counting Kit-8 (CCK8) (MeilunBio, Guangzhou, China) was employed to assess cell viability 48 h following the addition of oxaliplatin. The growth-inhibitory curves of oxaliplatin-resistant HGC-27 cells versus the parental cells were drawn, and the IC50 of oxaliplatin of each cell line was calculated.

### 2.12 Colony-formation assay

The indicated cells (1000 cells/well) were plated into six-well plates and the plates were then cultured at 37°C for 10 to 14 days. After 15-min fixation with formaldehyde (10%), cells were stained with 1.0% crystal violet for 30 s before counting the colonies. Finally, cell clones (> 50 cells) were counted and analyzed.

### 2.13 Wound healing assay

For wound healing assay, cells were inoculated into 6-well plates for culture to form a confluent layer and wounds were created by scraping using sterile tips. The cells were then rinsed thoroughly with PBS and further cultured in serum-free medium. Images were acquired at 0 and 24 h with microscope and the rate of cell migration was obtained by Image J.

### 2.14 Apoptosis assay

For apoptosis analysis, the cells were seeded into plates of six wells and then treated with oxaliplatin (Aladdin, Shanghai) for 24 h; afterward, they were digested by trypsin without addition of EDTA and washed for two times using PBS; after addition of AnnexinV- FITC and PI staining solution, they were incubated at ambient temperature in dark place for 15 min. Then, the apoptotic rate was determined with use of the flow cytometry.

### 2.15 Patients and tissue samples

We collected primary gastric cancer tissues and adjacent non-tumor tissues from 14 gastric cancer patients who underwent radical gastrectomy at The First Affiliated Hospital of Sun Yat-sen University between 2010 and 2017, and from another 14 patients who received the same procedure at The Seventh Affiliated Hospital of Sun Yat-sen University from 2021 to 2024. Clinicopathological features were gathered for patients undergoing radical gastrectomy, all of whom had not received any radiotherapy or chemotherapy before surgery. Tumor stage**s** were classified based on the American Joint Committee on Cancer (AJCC) TNM staging system. This study received approval from the Ethics Committee of The First Affiliated Hospital of Sun Yat-sen University (No.KY-2020–024-01) and The Seventh Affiliated Hospital of Sun Yat-sen University (No.KY-2021–072-01), and all participants provided written informed consent.

### 2.16 Immunohistochemistry

Immunohistochemical analysis of DCLK1 expression was conducted in paraffin-embedded gastric cancer specimens. In brief, paraffin-embedded slices were subjected to heating at 65°C for 2 h. Tissue sections were sequentially dewaxed and rehydrated through immersion in dimethylbenzene followed by a descending ethanol series. Following antigen retrieval in a microwave oven with an EDTA solution for 15 minutes, the slices were cooled at room temperature. After washing with PBS, the slices were treated with H_2_O_2_ to deactivate endogenous catalase. Subsequently, the sections were blocked in 5% goat serum for 30 min and incubated with primary antibody against DCLK1 (Proteintech, 21699-1-AP) overnight at a temperature of 4 °C. The next day, the slices were incubated with secondary antibody for 30 min at room temperature. Next, we performed color development using the DAB Detection Kit (Gene Tech, #GK600511) according to the manufacturer’s instructions. The slices were photographed and recorded by the Whole Slide Scanner VS200 (Olympus). Protein expression was assessed and verified by two independent pathologists based on staining intensity and the proportion of positively stained cells.

### 2.17 Statistical analysis

All statistical analyses were completed using Prism (Version 10.1) and R (Version 4.2.1) software.

One-way analysis of variance (ANOVA) and unpaired Student’s t-test were adopted to assess differences between groups. A two-tailed P < 0.05 was set to determine statistical significance.

## 3 Results

### 3.1 STAD classification pattern based on histone acetylation-related genes

**Figure 1:**
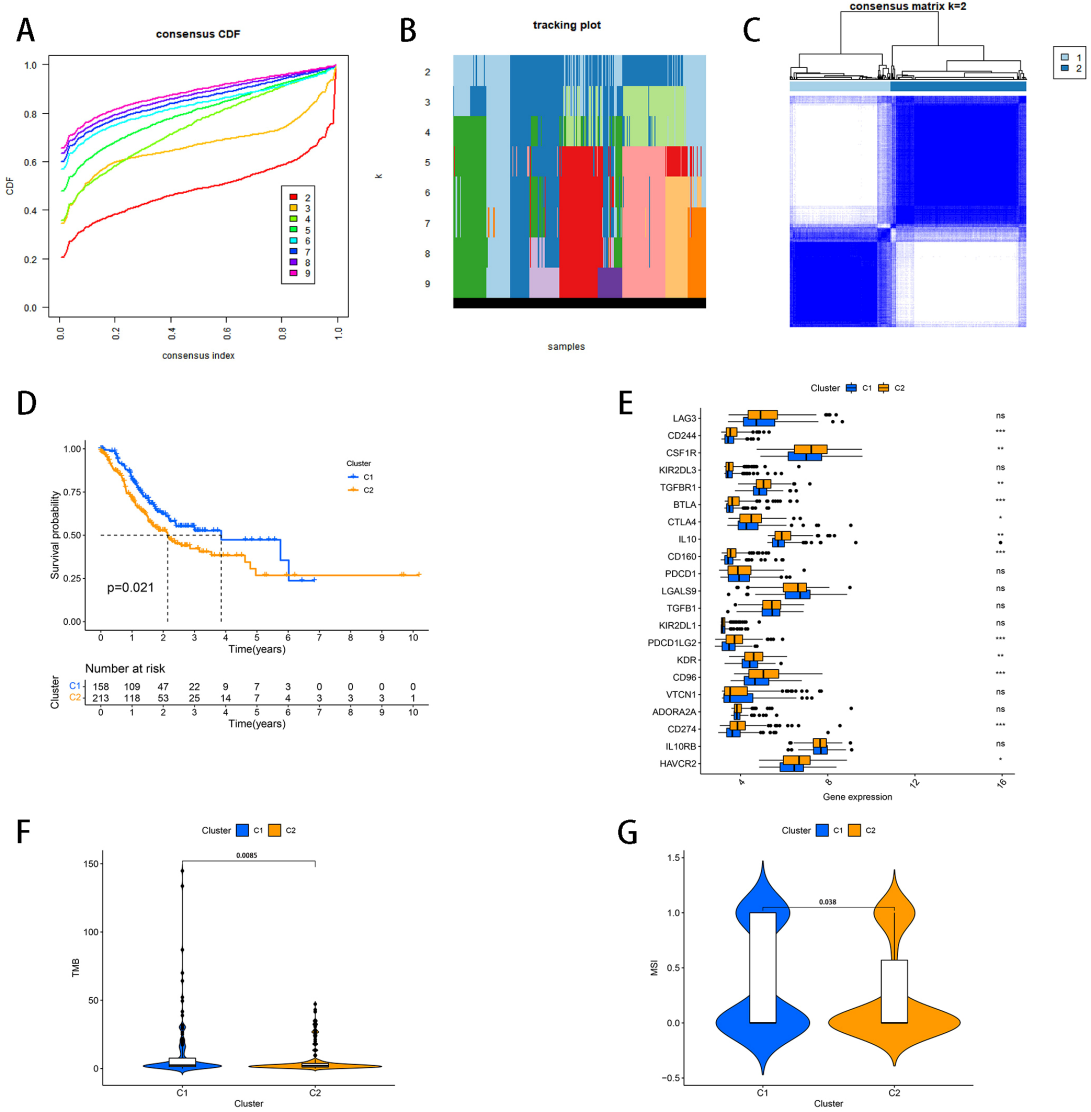
STAD classification pattern based on histone acetylation−related genes. **(A)** CDF curves **(B)** Tracking Plot **(C)** STAD patients were divided into two groups according to the consensus clustering matrix (K = 2). **(D)** Kaplan–Meier OS curves for the two clusters. **(E)** Expression of immune checkpoint-related genes between two clusters. **(F)** Difference of TMB between two clusters **(G)** Difference of MSI between two clusters. *represents p < 0.05; **represents p < 0.01; ***represents p < 0.001; ns represents p ≥ 0.05.

To analyze the connections between the histone acetylation-related genes and STAD, consensus clustering analysis was performed in the TCGA-STAD cohort for STAD patients. The results of the CDF curves and tracking plot showed that the optimal cluster number was K = 2 (**Figures 1A, B**). **Figure 1C** demonstrated that the maximum intra-group correlations with low inter-group correlations were observed when K = 2 and STAD patients could be split into two clusters according to differential expression patterns. K-M analysis was performed for comparison of overall survival advantage between two clusters. As indicated in **Figure 1D**, the survival probability was higher in cluster 1 (C1) than in cluster 2 (C2) (P= 0.021). Then, the immune checkpoint-related genes between the two clusters were examined with respect to their expression, and there were 12 DEGs (CD244, CSF1R, TGFBR1, BTLA, CTLA4, IL10, CD160, PDCD1LG2, KDR, CD96, CD274 and HAVCR2) between C1 and C2 (**Figure 1E**). These 12 genes showed higher expression levels in C2 (**Figure 1E**). In addition, we investigated the difference of TMB and MSI between two clusters and results indicated that C1 had higher TMB and MSI than C2 (**Figures 1F, G**).

**Figure 2:**
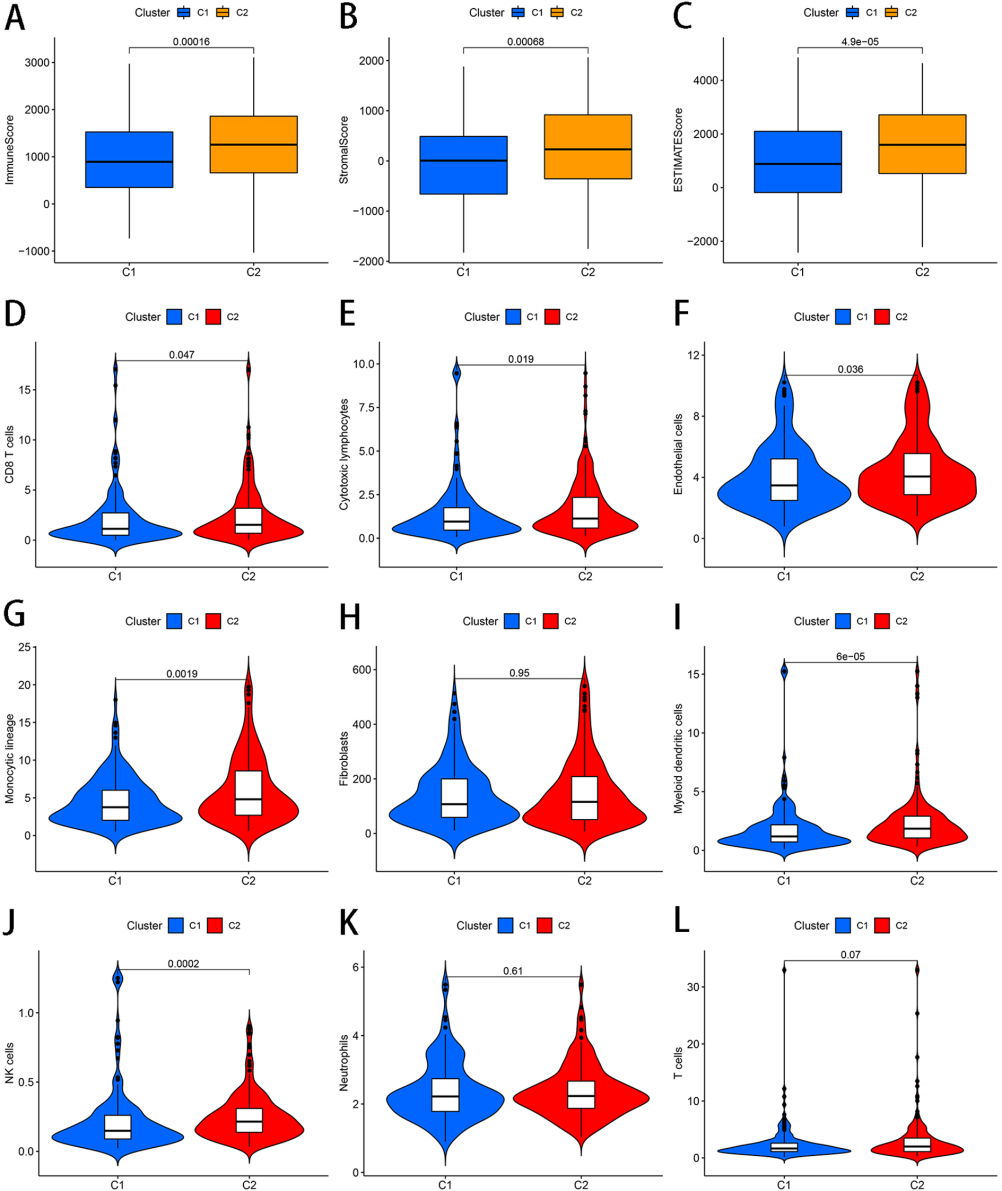
The relationship between two clusters and different immune cell infiltration. **(A)** Immune Score **(B)** Stromal Score **(C)** ESTIMATE Score **(D)** CD8 T cells **(E)** Cytotoxic lymphocytes **(F)** Endothelial cells **(G)** Monocytic lineage **(H)** Fibroblasts **(I)** Myeloid dendritic cells **(J)** NK cells **(K)** Neutrophils **(L)** T cells.

We also analyzed different immune cell infiltration level between C1 and C2. The results in **Figures 2A–C** showed that C2 have higher ImmuneScore, StromalScore and ESTIMATEScore (**Figures 2A–C**). Furthermore, we found that significant differences exist in some immune cell types including CD8 T cells, Cytotoxic lymphocytes, Endothelial cells, Monocytic lineage, Myeloid dendritic cells and NK cells with a p value <0.05 for all, specifically, 0.047, 0.019, 0.036, 0.0019, 6e-05 and 0.0002, respectively, and C2 had more these types of immune cell (**Figures 2D–L**).

### 3.2 Construction of eight-gene prognostic model

**Figure 3:**
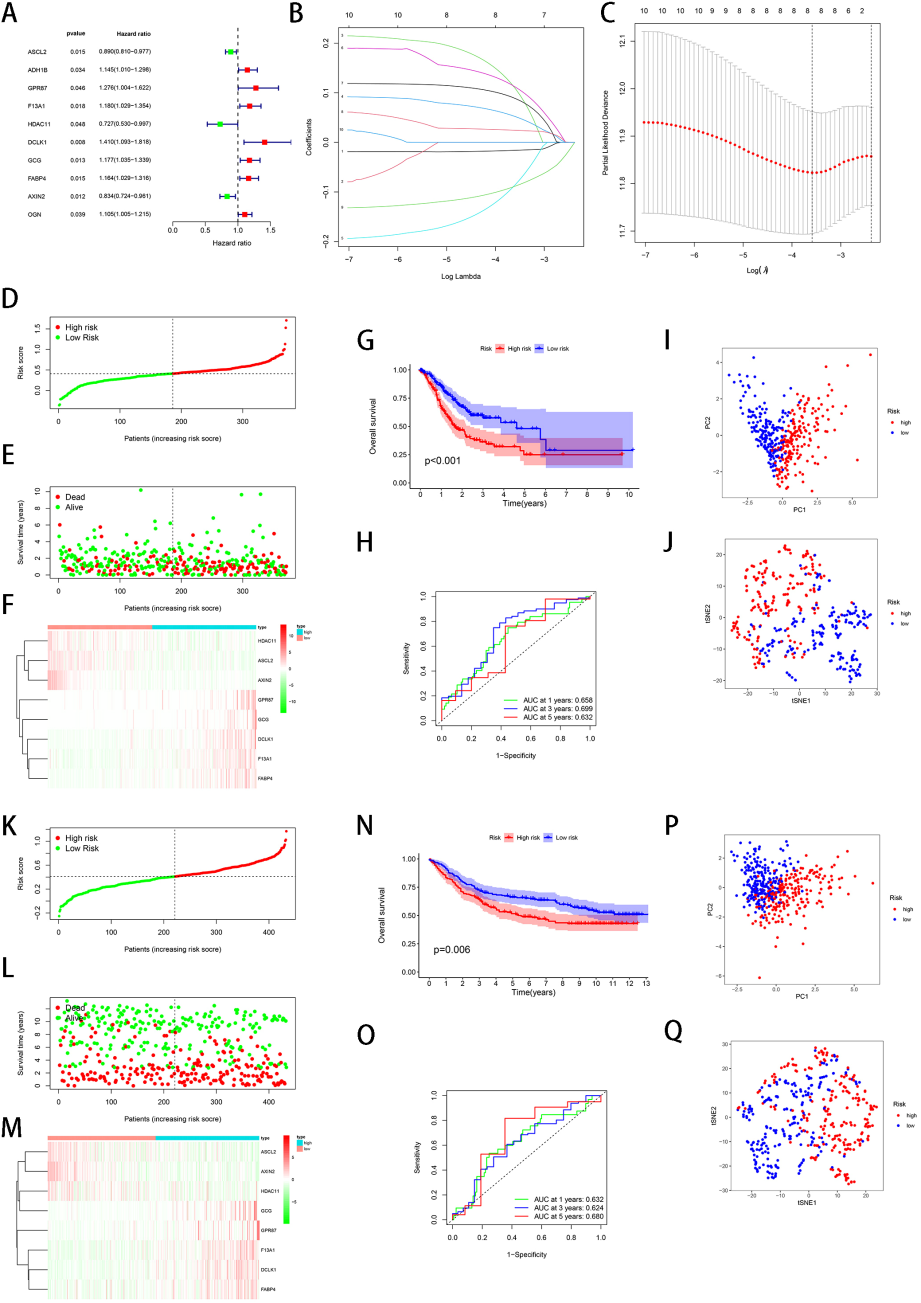
Construction of histone acetylation-related gene prognostic model based on the STAD-TCGA cohort and validation of the risk signature based on GSE84437 dataset. **(A)** Univariate cox regression analysis revealed 10 acetylation-related genes correlated with prognosis. **(B)** Acetylation-related genes were penalized by LASSO Cox regression analysis. **(C)** Cross-validation for tuning parameter selection in the LASSO Cox regression. **(D)** Distribution of STAD-TCGA patients based on the risk score. **(E)** The survival status of each STAD-TCGA sample based on the risk score. **(F)** The expression patterns of 8 prognostic genes between high and low risk groups in STAD-TCGA. **(G)** Kaplan–Meier OS curves of patients in the high- and low-risk groups. **(H)** ROC curves **(I)** Principal component analysis (PCA) plot for STAD upon the basis of risk score. **(J)** t-SNE analysis **(K)** Distribution of STAD patients in GSE84437 dataset based on the risk score. **(L)** The survival status of each STAD patients in GSE84437 dataset **(M)** The expression patterns of 8 prognostic genes between high and low risk groups in GSE84437 dataset. **(N)** Kaplan–Meier OS curves of patients in the high and low-risk groups. **(O)** ROC curves **(P)** PCA plot for STAD upon the basis of risk score. **(Q)** t-SNE analysis.

We screened out the differentially expressed genes between C1 and C2 through the “limma” package. Then prognostic significance of histone acetylation-related DEGs was assessed by using univariate Cox regression analyses. The 10 genes (ASCL2, ADH1B, GPR87, F13A1, HDAC11, DCLK1, GCG, FABP4, AXIN2, OGN) were retained for subsequent analysis (**Figure 3A**). Using LASSO Cox regression, a signature of eight genes was generated based on optimum λ value (**Figures 3B, C**). These 8 genes (ASCL2, GPR87, F13A1, HDAC11, DCLK1, GCG, FABP4, AXIN2) were regarded as prognostic factors for STAD, and for which the calculation of risk score was: risk score = (−0.016*ASCL2 exp.) + (0.108*GPR87 exp.) + (0.052*F13A1 exp.) + (−0.085*HDAC11 exp.) + (0.111*DCLK1 exp.) + (0.088*GCG exp.) + (0.024*FABP4 exp.) + (−0.088*AXIN2 exp.). In addition, division of STAD patients was conducted as two groups of high- and low-risk on the basis of their median risk score (**Figure 3D**). Patients with STAD in the high-risk one possessed a greater risk of death and less survival time (**Figure 3E**). The expression patterns of these eight prognostic genes between the groups were demonstrated by heatmap (**Figure 3F**). Patients with STAD in the high-risk group presented a lower overall survival (OS) than those in the low-risk group (**Figure 3G**). **Figure 3H** indicated that the area under the ROC curve was 0.658, 0.699 and 0.632, respectively, at 1 year, 3 years and 5 years. The PCA and t-SNE analysis suggested that patients with varying risk levels in STAD can be effectively divided into two directions (**Figures 3I, J**).

### 3.3 Validation of the risk signature

The GSE84437 dataset was used for validation of robustness of our signature. Similar to the previously described results, STAD patients in GSE84437 dataset were also assigned into two groups of low- and high-risk in light of the median risk score (**Figure 3K**). The STAD patients with different risks could be assigned into two directions, which was confirmed by PCA and t-SNE analysis (**Figures 3P, Q**). The patients showed a greater risk of death, less survival time and decreased OS in the high-risk group than those in the low-risk group (**Figures 3L, N**). The expression patterns of eight prognostic genes between the two groups were shown by heatmap (**Figure 3M**). The area under the ROC curve reached 0.632 at 1 year, 0.624 at 3 years, and 0.680 at 5 years (**Figure 3O**).

### 3.4 Independent prognostic value of the risk signature

**Figure 4:**
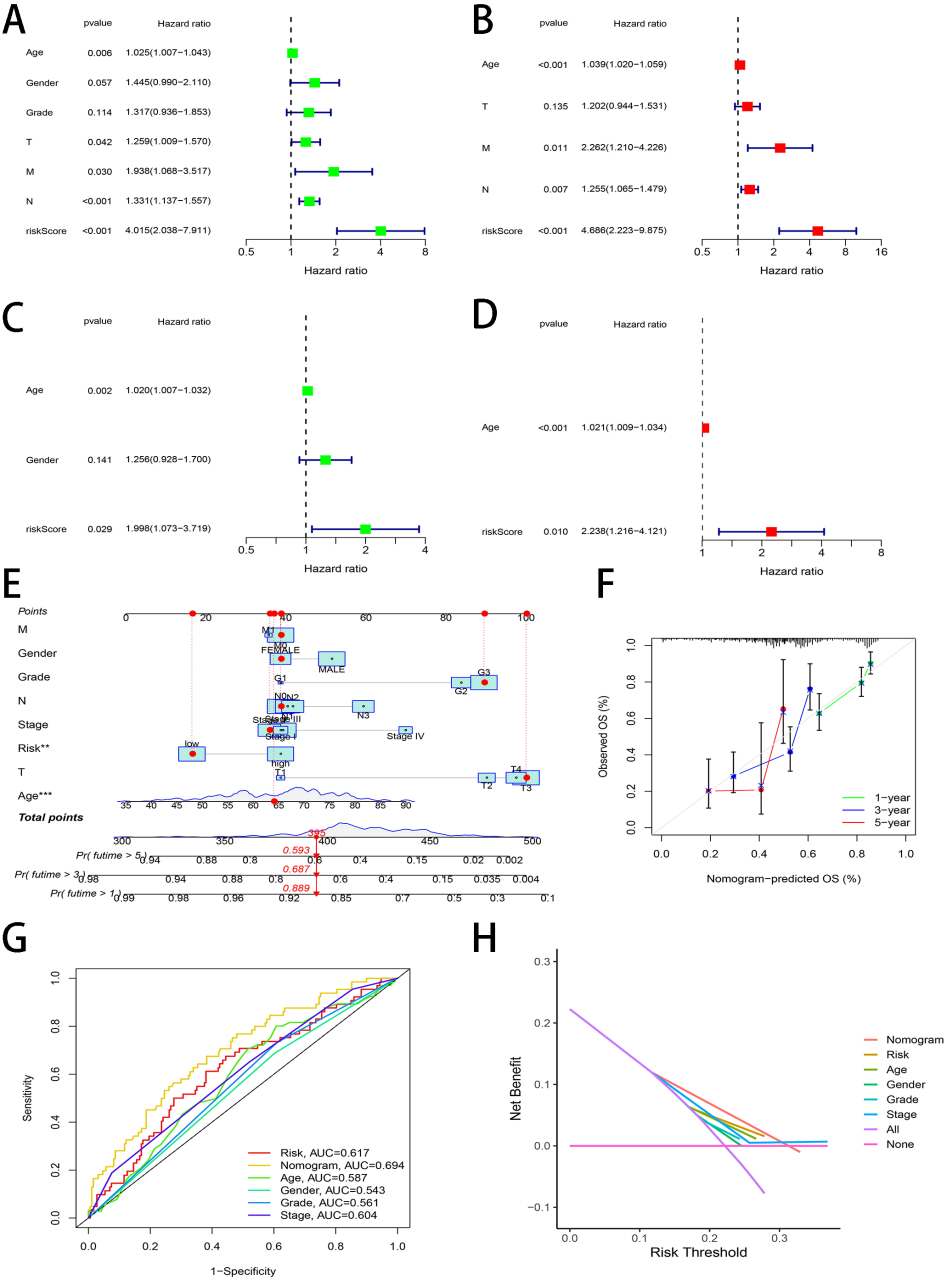
Independent prognostic value of the risk signature and construction of a prognostic nomogram. **(A)** The forest plot of univariate analysis for the TCGA cohort **(B)** The forest plot of multivariate analysis for the TCGA cohort **(C)** The forest plot of univariate analysis for the GEO cohort **(D)** The forest plot of multivariate analysis for the GEO cohort **(E)** Nomogram of the TCGA cohort predicting the survival rate **(F)** Calibration plot analysis to evaluate nomogram accuracy **(G)** The AUC values of the nomogram and clinicopathological features **(H)** The decision curve analyses plot.

To determine the feasibility of this novel gene signature being used as a prognostic predictor independent of other factors, the Cox regression of univariate and multivariate was carried out. For the univariate analysis, we discovered that the identification of risk score as an independent prognostic predictor for STAD patients was demonstrated in both the TCGA cohort (HR = 4.015, 95% CI: 2.038–7.911, **Figure 4A**) and GEO cohort (HR = 1.998, 95% CI: 1.073–3.719, **Figure 4C**). For the multivariate analysis similar results were found, indicating that the risk score is an independent prognostic predictor (TCGA cohort: HR = 4.686, 95% CI: 2.223–9.875, **Figure 4B**; GEO cohort: HR= 2.238, 95% CI: 1.216–4.121, **Figure 4D**). A nomogram was established to predict 1, 3, and 5-year OS in TCGA-STAD cohort (**Figure 4E**). The calibration curve confirmed that the nomogram operated with an extremely high consistency with an ideal model (**Figure 4F**). The results of the ROC curves showed that the nomogram had the largest AUC (AUC=0.694) than other clinicopathological features (**Figure 4G**). Decision curves of **Figure 4H** also showed the nomogram presented larger net benefits.

### 3.5 Correlations between somatic mutation, immune subtype, clinicopathological features and risk score

**Figure 5:**
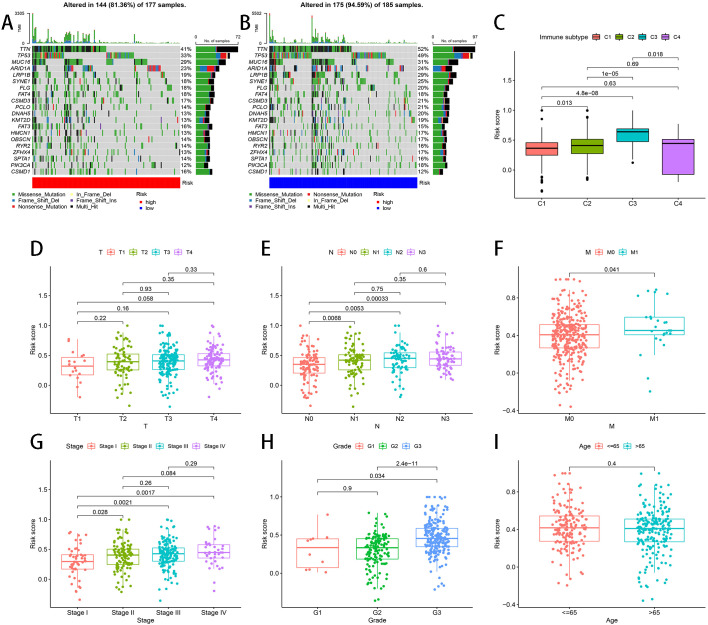
Associations between risk score and somatic mutation, immune subtype and clinicopathological features. **(A)** somatic mutation landscape in high-risk group **(B)** somatic mutation landscape in low-risk group **(C)** Relationship between risk score and immune subtypes. **(D-I)** The relationship between risk score and T stage **(D)**, lymph node metastasis **(E)**, distant metastasis **(F)**, clinical stage **(G)**, tumor grade **(H)** and age **(I)**.

**Figure 6:**
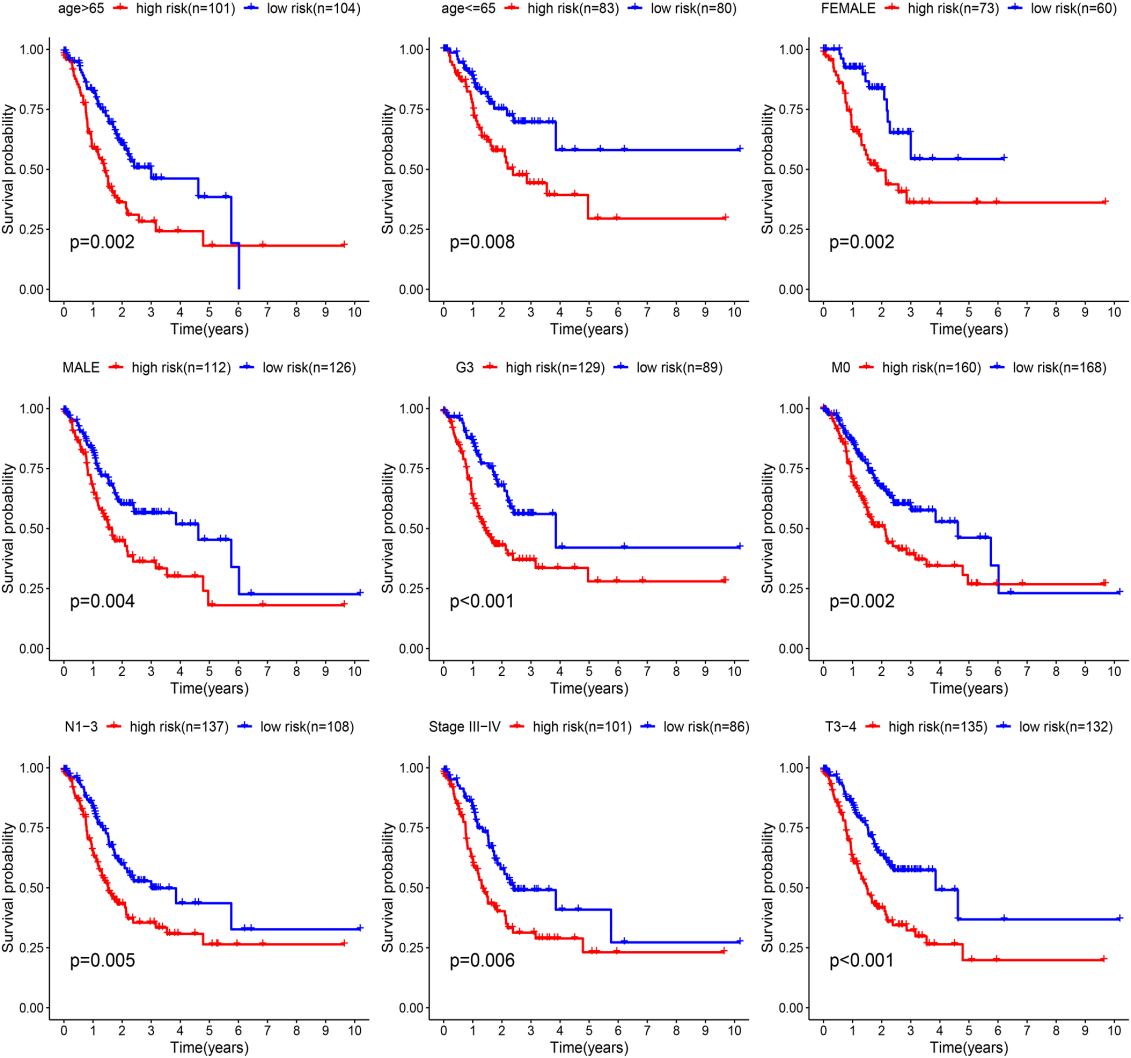
The KM curves difference of two risk groups in age >=65, age<= 65, Female, Male, G3, M0, N1-3, Stage III-IV and T3-4.

We investigated different somatic mutation landscapes between TCGA-STAD patients defined as high- and low-risk (**Figures 5A, B**). Patients in the low-risk group had higher mutation rates than the high-risk group (94.59 vs 81.36%). In terms of gene mutation frequency, TTN, TP53 and MUC16 were the most altered gene. The most frequent mutation was missense mutation in patients with STAD. According to **Figure 5C**, risk score and different immune subtypes were conspicuously correlated with each other. In addition, a positive correlation was also present between the risk score and lymph node metastasis, distant metastasis, clinical stage and tumor grade except for age and T stage (**Figures 5D–I**). The K-M curves implied that high-risk patients exhibited a less favorable prognosis than those classified as low-risk in age >65, age<= 65, Female, Male, G3, M0, N1-3, Stage III-IV and T3-4 (**Figure 6**).

### 3.6 Relationships between risk score and tumor microenvironment, immune cell infiltration and immune activity

**Figure 7:**
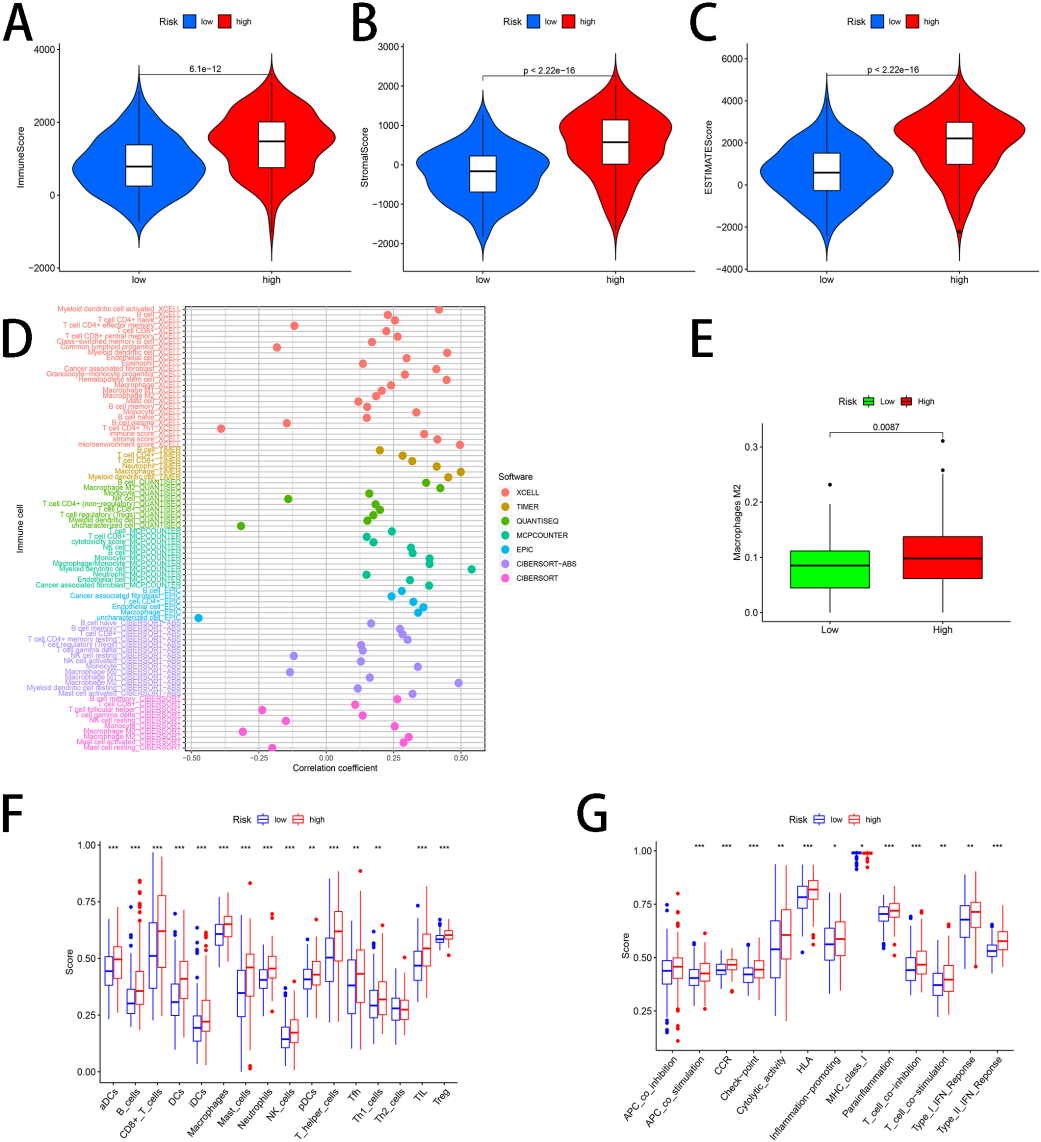
Relationships between risk score and tumor microenvironment, immune cell infiltration and immune activity. **(A)** Immune score **(B)** Stromal score **(C)** ESTIMATE score **(D)** correlations between tumor-infiltrating immune cells and risk score based on 7 known algorithms **(E)** Different macrophages M2 level in two subgroups **(F)** The difference in enrichment scores of different types of immune cells between two risk subgroups. **(G)** The difference in enrichment scores of different types of immune pathways between two risk subgroups. *represents p < 0.05; **represents p < 0.01; ***represents p < 0.001.

The relationships between risk score and TME were determined, using calculations of Immune, Stromal and ESTIMATE scores. As shown in **Figures 7A–C**, the Immune, Stromal and ESTIMATE scores were higher in the high-risk group. Based on seven known algorithms, the correlation of tumor-infiltrating immune cells to the risk score was analyzed (**Figure 7D**). Since tumor-associated macrophages (TAMs) are critical in TME, we explored the relationships between our risk score and TAMs. **Figure 7E** implied that the level of M2 macrophages was higher in the high-risk group than in the low-risk group. Furthermore, results of functional analysis suggested that our histone acetylation-related gene signature was notably associated with immune activity and the high-risk subgroup exhibited elevated infiltrating levels of most immune cells except for Th2_cells (**Figure 7F**). **Figure 7G** proved that the high-risk group showed more activity for most immune pathways excepting MHC_class_I, which showed less activity. The activity of APC_co_inhibition pathway revealed no significant difference between two subgroups (**Figure 7G**).

### 3.7 Immunotherapy prediction

**Figure 8:**
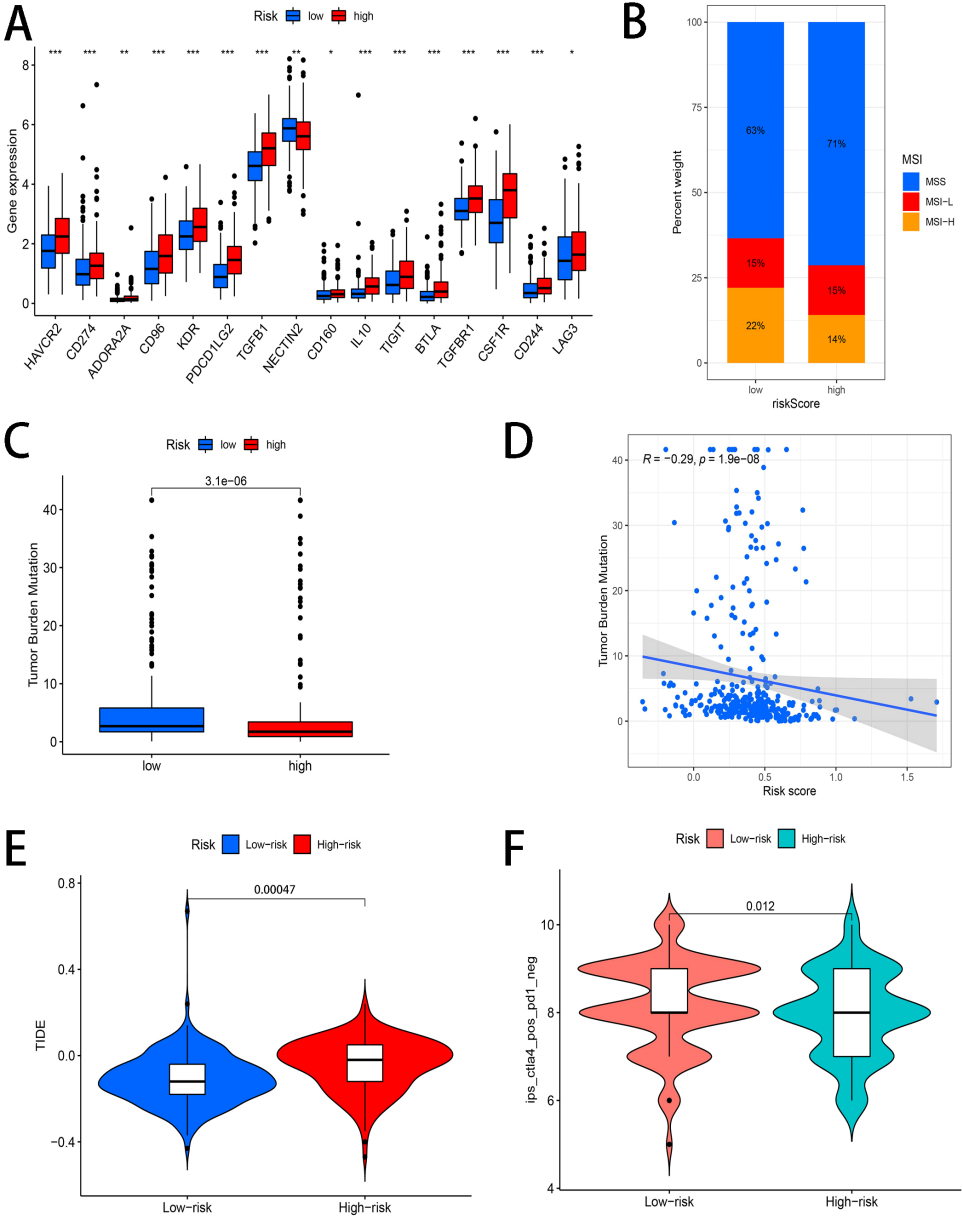
Immunotherapy prediction **(A)** Different expression of immune checkpoint-related genes between two risk subgroups. **(B)** Different MSI status (MSS, MSI-H and MSI-L) between two subgroups. **(C)** The difference of TMB was significant in STAD patients with different risk. **(D)** The correlations between risk score and TMB. **(E)** The correlations between risk score and TIDE score **(F)** Differences in immunophenoscores between patients in two risk groups received CTLA4 inhibitor. *represents p<0.05; **represents p<0.01; ***represents p<0.001; ns represents p≥0.05.

As shown in **Figure 8A**, 15 immune checkpoint-related genes (HAVCR2, CD274, ADORA2A, CD96, KDR, PDCD1LG2, TGFB1, CD160, IL10, TIGHT, BTLA, TGFBR1, CSF1R, CD244 and LAG3) were conspicuously overexpressed in the high-risk group, excepting one immune checkpoint-related gene (NECTIN2), which was obviously overexpressed in the low-risk group. Then, we investigated the different MSI status (MSS, MSI-H and MSI-L) between two subgroups. The data suggested that the MSI of high-risk group is lower (**Figure 8B**). We also observed that in the high-risk group, the TMB was lower, and there was a negative correlation between risk score and TMB (R=-0.29, P=1.9e-08) (**Figures 8C, D**). **Figure 8E** shows that the high-risk group had remarkably high TIDE scores than the low-risk group through the use of the TIDE algorithm, indicating that the low-risk group may have more immunotherapeutic effects than the high-risk group. Furthermore, TCIA immunotherapy prediction showed that the low-risk patients had a superior response to CTLA4 inhibitor (**Figure 8F**).

### 3.8 Drug sensitivity based on the prognostic signature

**Figure 9:**
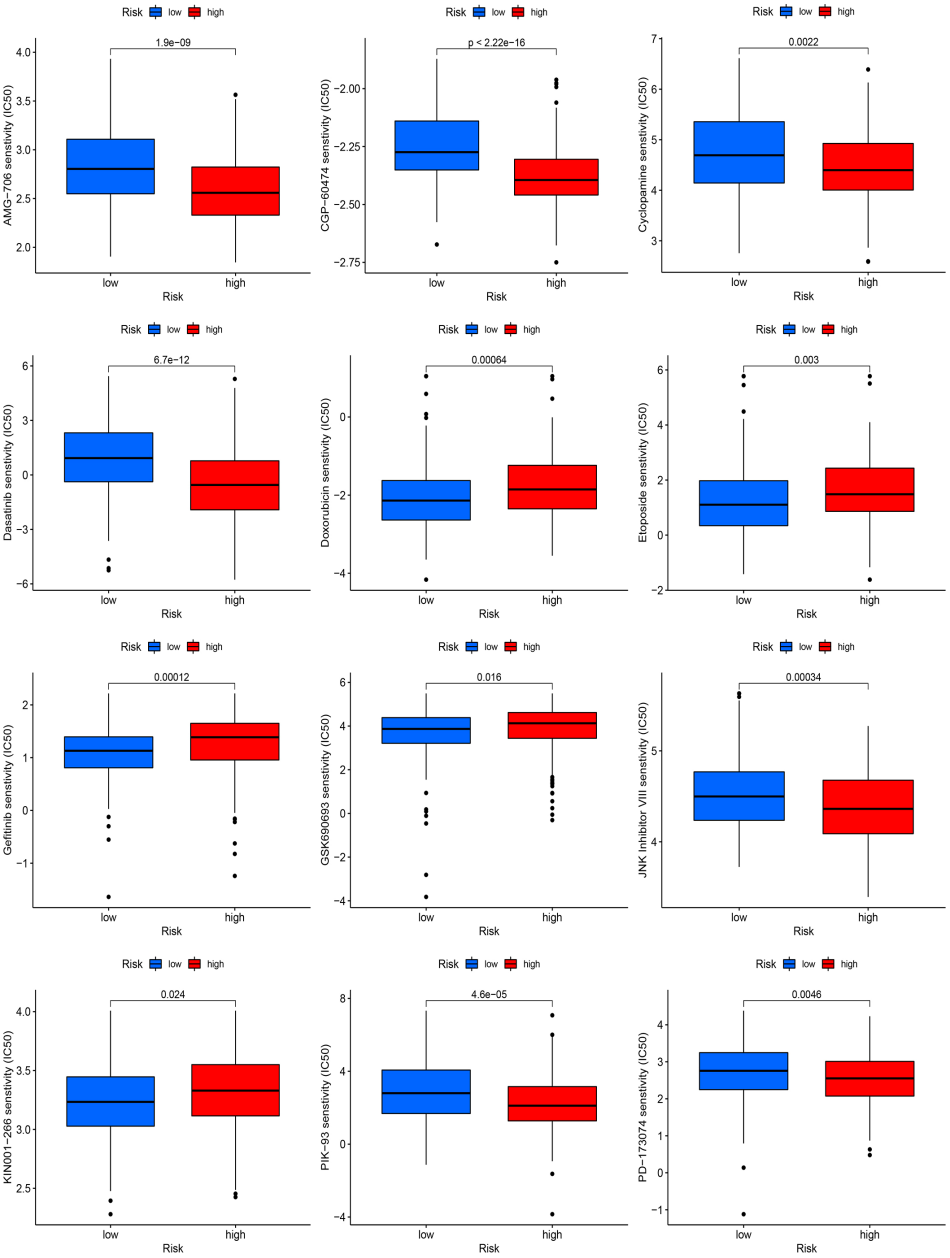
Drug sensitivity based on the prognostic signature.

IC50 was used to estimate the difference between the two risk subgroups for 12 drugs. Our findings observed that patients from the high-risk group showed a higher estimated IC50 for five drugs including Doxorubicin, Etoposide, Gefitinib, GSK690693 and KIN001–266 but showed a lower estimated IC50 for the remaining seven drugs including AMG-706, CGP-60474, Cyclopamine, Dasatinib, JNK Inhibitor VIII, PIK-93 and PD-173074 (**Figure 9**).

### 3.9 Functional analyses

**Figure 10:**
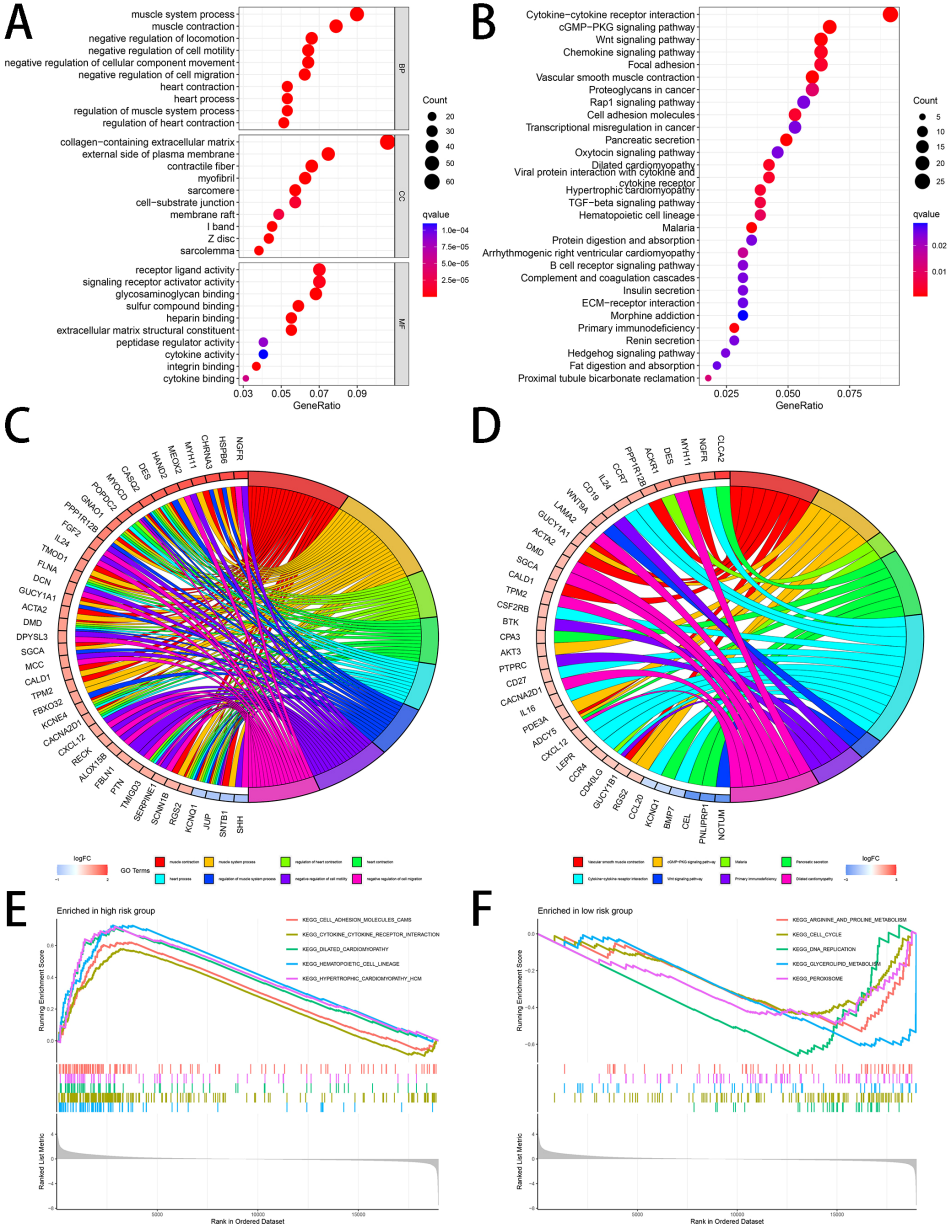
Functional Analyses **(A, C)** GO enrichment analysis **(B, D)** KEGG pathway analysis **(E, F)** GSEA analysis.

To analyze the differences between the two groups regarding gene functions and enrichment, we extracted DEGs between the two groups with use of R package (“limma”). With these DEGs, GO and KEGG analyses were performed, and as shown in **Figures 10A–D**. The results of GO analysis showed that the DEGs were related to muscle system process, etc. in BP, collagen-containing extracellular matrix, etc. in CC, receptor ligand activity, etc. in MF (**Figures 10A, C**). According to the data from KEGG, the DEGs were abundant in cytokine-cytokine receptor interaction, signaling pathways of cGMP-PKG and Wnt (**Figures 10B, D**). GSEA analysis was used to find which pathways were enriched in the two groups and the results are shown in **Figures 10E**, and **F**. The cell adhesion molecules cams, cytokine-cytokine receptor interaction and dilated cardiomyopathy were conspicuously enriched in the high-risk group. **Figure 10F** showed prominently enriched arginine and proline metabolism, cell cycle and DNA replication in the low-risk group.

### 3.10 Effects of DCLK1 on the proliferation, migration and oxaliplatin resistance of gastric cancer cells *in vitro* and clinical significance of DCLK1 in GC

**Figure 11:**
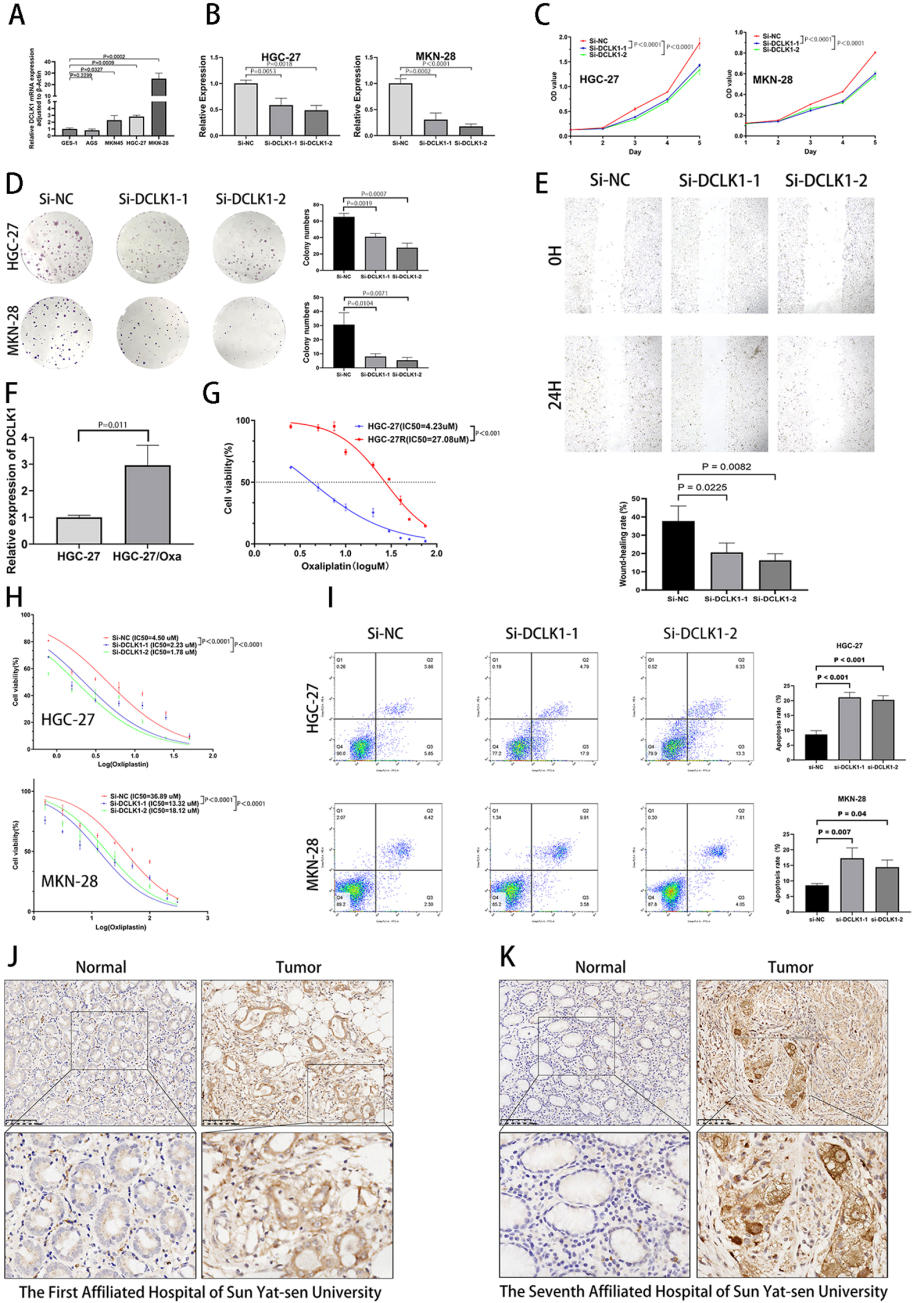
Biological function of DCLK1 in GC cells and oxaliplatin-resistant GC cells. **(A)** The mRNA expression of DCLK1 in GC cell lines (HGC-27 and MKN-28) and normal gastric epithelial cell line (GES-1) were assessed by qRT-PCR. **(B)** DCLK1 RNA expression in HGC-27 and MKN-28 transfected with Si-DCLK1-1, Si-DCLK1–2 and Si-NC were determined by qRT-PCR. **(C)** CCK-8 assays were performed to observe HGC-27 and MKN-28 cells viability after DCLK1 knockdown. **(D)** Colony formation assays were implemented in silenced DCLK1 gastric cancer cell lines (HGC-27 and MKN-28). **(E)** Wound−healing assays were conducted to confirm HGC-27 cell migration with DCLK1 knockdown. **(F)** DCLK1 expression in oxaliplatin-resistant HGC-27(HGC-27/Oxa) gastric cancer cells compared with their parental cells. **(G)** Cell viability of HGC-27 and HGC-27/Oxa by CCK8 assay. **(H)** Cell viability in HGC-27/Oxa and MKN-28/Oxa transfected with DCLK1 SiRNAs and Si-NC were confirmed by CCK-8 assay. **(I)** Apoptotic ratio of HGC-27/Oxa and MKN-28/Oxa cells transfected with DCLK1 SiRNAs and Si-NC were estimated by Flow cytometry. Data was presented as mean ± SD. **(J)** Representative IHC images of DCLK1 in gastric cancer tissues and corresponding normal tissues (The First Affiliated Hospital of Sun Yat-sen University). Scale bar, 100 μm. **(K)** Representative IHC images of DCLK1 in gastric cancer tissues and corresponding normal tissues (The Seventh Affiliated Hospital of Sun Yat-sen University). Scale bar, 100 μm.

Among the eight histone acetylation-related genes, DCLK1 had the largest correlation coefficient in the prognostic model. Therefore, we carried out *in vitro* analysis to determine the functions of DCLK1 in GC cells. The RT-qPCR experimental results suggested that the expression of DCLK1 was significantly higher in MKN-45, MKN-28 and HGC-27 of GC cell lines than in the normal human gastric epithelial cell line GES-1 (**Figure 11A**). DCLK1 exhibited the highest expression levels in HGC-27 and MKN-28. Therefore, these two GC cell lines were selected for subsequent studies. To determine whether DCLK1 affected progression in gastric cancer cells, we designed siRNA to silence DCLK1 expression in MKN-28 and HGC-27 cells. The efficiency of DCLK1 silencing in MKN-28 and HGC-27 was confirmed through RT-qPCR (**Figure 11B**). Following the successful gene knockdown demonstrated previously, the CCK-8 assay was performed to validate the effects of DCLK1 on the proliferation and viability of MKN-28 and HGC-27 cells. The results proved that the proliferation of these two gastric cancer cell lines were impaired after knocking down DCLK1 compared to the Si-NC group (**Figure 11C**). Furthermore, colony formation experiments showed that colony formation was lower in the DCLK1 down-regulation group (**Figure 11D**). **Figure 11E** also manifested that the migration of HGC-27 was weakened in si-DCLK1 groups. Since oxaliplatin is commonly used as a first-line chemotherapeutic agent for gastric cancer, we proceeded to investigate the impact of DCLK1 expression on the effectiveness of this chemotherapy drug. To check the DCLK1 expression in Oxaliplatin (Oxa)-resistant GC cells, RT-PCR was performed (HGC-27/Oxa), and the findings manifested that DCLK1 was more expressed in Oxa-resistant GC cells (HGC-27/Oxa) than in the parental ones (**Figure 11F**). When treated with increasing levels of Oxaliplatin, HGC-27/Oxa cells exhibited greater viability (**Figure 11G**). In addition, when comparing the DCLK1 knockdown transfection group to the Si-NC group, the 50% maximal inhibitory concentration (IC50) value of oxaliplatin was lower in si-DCLK1 group (**Figure 11H**). The apoptotic rate of HGC-27 and MKN-28 cells treated with Oxaliplatin increased through the suppression of DCLK1 (**Figure 11I**). In summary, the knockdown of DCLK1 could repress gastric cancer cell in terms of cell proliferation, migration and oxaliplatin resistance. To investigate the clinical relevance of DCLK1 in GC, IHC analysis was performed on paraffin-embedded gastric cancer tissue sections from 14 patients who underwent radical gastrectomy at each of two different hospitals (The First Affiliated Hospital of Sun Yat-sen University and The Seventh Affiliated Hospital of Sun Yat-sen University). IHC staining results of tissue sections from GC patients in these two hospitals revealed that DCLK1 expression in gastric cancer tissues was higher than in normal gastric tissues (**Figures 11J, K**). In addition, elevated DCLK1 expression was associated with Borrmann’s Type, depth of invasion, lymph node metastasis, and TNM stage in GC patients (**Supplementary Table 5**).

## 4 Discussion

Stomach adenocarcinoma (STAD) accounts for the most gastric cancer cases. Current research indicates that histone acetylation is a pivotal factor in the occurrence and progression of STAD. Deng et al. suggested that over-expression of EGFL7 were found in gastric cancer cell lines and the potential mechanism was the hanging of histone acetylation levels in the EGFL7 promoter caused by MALAT1 (31). Yamamura’s research demonstrated that hyperacetylated status occurred in histones H3 and H4 in the GATA4-positive gastric cancer cells, using CHIP assay (32). Wisnieski et al. found that BMP8B had increased acetylated H3K9 and H4K16 levels in gastric cancer and abnormal expression of BMP8B was related to poorly differentiated gastric cancer (33). However, current research generally focuses on the relationship between histone acetylation level of single or several genes and STAD, and neglects a systematic development of a prognostic model to explore the associations between histone acetylation-related genes and STAD. In addition, histone methylation, another important type of histone modification, has been extensively studied, whereas research on histone acetylation is still not thorough enough. Therefore, a model derived from multiple histone acetylation-related genes is urgently required for STAD patients.

In our study, we first used consensus clustering analysis to determine two molecular subtypes based on 40 known histone acetylation-related genes (34). The findings indicated that C2 had worse prognosis, lower TMB and MSI, higher and immune cell infiltration level versus C1. Then, we screened out DEGs between C1 and C2 and filtered out prognostic significance of these DEGs. Through utilization of the LASSO Cox regression model, we generated a signature composed of eight genes (ASCL2, GPR87, F13A1, HDAC11, DCLK1, GCG, FABP4, AXIN2). ASCL2 (Achaete scute-like 2) belongs to the basic helix-loop-helix (BHLH) family of transcription factors (35). As an indispensable downstream element of Wnt/β-catenin signaling pathway, ASCL2 is closely associated with the occurrence and development of different cancers (36). ASCL2 was an independent indicator in recurrent breast cancer patients, which can also estimate the risk of relapse in breast cancer (37). ZUO et al. found that ASCL2 affected the process of metastasis in gastric cancer by regulating miR223 expression (38). GPCRs (G protein-coupled receptors), a member of membrane signaling proteins in eukaryote, are encoded by about 800 genes and participate in different diseases (39, 40). GPR87 (G protein-coupled receptor 87) is one of the GPCR family and is in the chromosome 3q24 (41). Recent studies have confirmed that GPR87 is overexpressed in several malignancies such as pancreatic cancer (42), lung cancer (43) and bladder cancer (44). Bai’s research proved that GPR87 might participate in tumorigenesis and progression of lung cancer, which may also be related to immune infiltration (45).One study detected that GPR87, as an oncogene, is expressed in pancreatic cancer cells and promotes cancer proliferation and metastasis by driving the NF-κB signaling pathway (42). F13A1 encodes coagulation factor XIII A subunit. This subunit has catalytic functions and acts as a plasma carrier molecule (46). Research has demonstrated the presence of abnormal expression of F13A1 in tumors. In bladder cancer, over-expression of F13A1 was correlated with worse prognoses (47). HDACs (histone deacetylases) are epigenetic regulators that are recruited by co-repressors or transcriptional complexes to gene promoters and participate in the regulation of gene transcription (48). HDACs are grouped into four principal classes, among which, HDAC11, the newly discovered HDAC enzyme, has been classified into Class IV HDAC and was regarded as a key factor in metabolism, obesity and immune functions (49–52). However, HDAC11 has been demonstrated to participate in tumor development and progression. The study from Bi et al. showed that HDAC11 can regulate the process of glycolysis and stemness of hepatocellular carcinoma through the LKB1/AMPK signaling pathway (53). A pan-cancer analysis indicated that HDAC11 is aberrantly expressed in multiple cancers and markedly correlated with survival outcomes of patients with different cancers (54). DCLK1 (doublecortin like kinase 1), which belongs to the protein kinase superfamily and the doublecortin family, is present on chromosome 13q13-q14.1 and was initially recognized as a crucial regulator of neurogenesis and neuronal migration (55). Recent research suggests that DCLK1 displays the characteristics of cancer stem cells (CSC) and was highly expressed in several cancers (56–60). Furthermore, research indicates that DCLK1 is essential for tumor progression, angiogenesis and epithelial-mesenchymal transition (61). GCG (glucagon) is a protein that is included in four distinct mature peptides (62). Research indicates that GCG and GLP-1 play a critical role in glucose metabolism and diabetes (63, 64). FABP4 belongs to the family of fatty acid-binding proteins (FABPs), which are small molecule proteins that can transport hydrophobic and bioactive fatty acids (65). Studies of FABP4 have focused on patient obesity, because of its aberrant expression in adipose tissue and differentiated adipocytes and macrophages (66, 67). Recent research has focused on the relationship between FABP4 and tumors. Tian et al. suggested that upregulated FABP4 facilitates the migration and invasion of colon cancer cells by facilitating FAs transport and activating AKT pathway and EMT (68). In addition, exogenous FABP4 played a vital role in breast cancer progression and regulated fatty acid transport proteins expression (69). The AXIN2 gene, located on chromosome 17q24, has been reported to have an indispensable role in Wnt signaling pathway (70, 71). Research has found abnormally expressed AXIN2 in cancers such as hepatocellular-cholangiocarcinoma, colon cancer and lung cancer (72–74).

After our histone acetylation-related gene signature was constructed, ROC analysis was conducted to confirm the prognostic signature with respect to the sensitivity and specificity. The signature enabled us to divide STAD-TCGA patients into two groups of high- and low-risk in light of their median risk score. We found that STAD patients in the high-risk group had significantly poorer survival outcomes than those in the low-risk group. Then, GEO database (GSE84437) was employed for validation of the risk signature. Furthermore, the results obtained from Cox regression of univariate and multivariable proved that our risk signature was a prognostic indicator independent from other factors for STAD patients. After that, a nomogram was constructed and calibration plots confirmed that this nomogram could accurately predict survival probability for patients with STAD.

Recently, mutations identification is regarded as a critical step in cancer risk assessment and some published research implied somatic mutation plays pivotal roles in STAD (75, 76). Therefore, we investigated the mutation landscape of our gene signature, and then found that the mutation rate is higher in the low-risk group than in the high-risk one and TTN, TP53 and MUC16 are the most altered gene. In addition, missense mutation was the most frequent mutation in patients with STAD. These findings offer novel indicators for research on how STAD and somatic mutations are correlated with each other and for the formulation of more precise treatments in those STAD patients with gene mutations. In addition, there was a positive correlation between the risk score and lymph node metastasis, distant metastasis, clinical stage and tumor grade except for age and T stage.

Tumor immunity is an important focus of cancer research, with some findings indicating strong relationships between histone acetylation and tumor immunity. Therefore, we examined the relationships between risk score and tumor microenvironment, immune cell infiltration and immune activity. Our findings suggested that the high-risk group had increased levels of infiltrating immune cells, enhanced activity of immune pathways and higher TME scores compared with the low-risk group. Tumor-associated macrophages (TAMs) were composed mostly of M0, M1 and M2 phenotypes, which have generally been found to promote cancer progression and development (77, 78). Research has defined M2 macrophages that play immunosuppressive and tumor promoting roles as TAMs. Our findings suggest that the level of macrophages M2 is higher in the high-risk group. This result could be a significant contributing factor to the unfavorable prognosis observed in the high-risk population. Cancer immunotherapy has made advances in the field of cancer treatments (79). Thus, we analyzed the immunotherapy prediction of our histone acetylation-related gene signature, and observed that high-risk group possessed lower MSI, lower TMB and higher TIDE scores. TCIA immunotherapy prediction revealed patients in the high-risk group had an inferior response to CTLA4 inhibitor. These findings confirmed that the low-risk group had a greater probability of responding effectively to immunotherapy than the high-risk group. The data regarding drug sensitivity provided the basis for the formulation of a more accurate treatment for STAD. We also performed functional analyses and found that DEGs in the two groups of high- and low-risk patients participated in cancer-related processes and pathways. For example, the Wnt signaling pathway and cGMP-PKG signaling pathway are strongly associated with the occurrence and progression of gastric cancer. Xiang et al. indicated that infection of H. pylori relies on ZEB1 to induce the over-expression of PRTG, which subsequently leads to the advancement of gastric cancer through activation of the cGMP/PKG signaling pathway (80). Research has shown that the Wnt pathway is closely related to gastric cancer and its dysregulation has been observed in almost half of GC cases (81).

Finally, DCLK1 was selected for further experimental investigation to explore the molecular mechanism of the model genes, based on its highest correlation coefficient. In this research, we verified that down-regulation of DCLK1 significantly suppressed proliferation, colony forming ability, migration, and oxaliplatin resistance. This finding indicated that DCLK1 is a cancer-promoting factor in gastric cancer and can also promote oxaliplatin resistance in gastric cancer cells.

Our study has some limitations. First, the main source of our data was publicly available datasets and further in-depth research, and *in vivo* experiments may be required for validation of our results. Second, larger and multicentric clinical samples and other clinical features (e.g., smoking, family background, BMI) must be included in such research. Finally, the specific mechanisms that determine histone acetylation regulation of STAD must be examined.

## 5 Conclusions

A novel histone acetylation-related gene signature, which possesses potential value in predicting the prognosis and immunotherapy effectiveness regarding STAD patients, was developed. This signature may serve as a reliable biomarker for prognosis of STAD and promote the identification of novel treatment targets for STAD. Furthermore, DCLK1 exhibited oncogenic roles and may act as a new target for STAD therapy.

## Data Availability

The original contributions presented in the study are included in the article/**Supplementary Material**. Further inquiries can be directed to the corresponding authors.
