# Supplementary material for: The histone acetylation-related gene signature predicts prognosis and immunotherapy response in stomach adenocarcinoma
Source: Front Oncol. 2025 Sep 2;15:1527253. doi: 10.3389/fonc.2025.1527253 (PMC12436397; doi:10.3389/fonc.2025.1527253)
Supplement: Supplementary file 1 [file Table1.docx]

| **Supplementary Table 1.** Differential genes between two clusters | | | | | |
| --- | --- | --- | --- | --- | --- |
| gene | Mean1 | Mean2 | logFC | pValue | FDR |
| ASCL2 | 36.90252883 | 12.00613423 | -1.619947981 | 1.37E-07 | 3.79E-06 |
| CSTA | 10.58016804 | 32.71951023 | 1.628788611 | 1.77E-06 | 2.77E-05 |
| LIPF | 109.8347722 | 420.7002723 | 1.937457888 | 0.006839239 | 0.019001527 |
| GKN1 | 95.6051533 | 425.7999714 | 2.155015564 | 0.00433559 | 0.013196898 |
| ADH1B | 2.436960374 | 5.002633148 | 1.037604868 | 0.001221669 | 0.004862129 |
| DPEP3 | 0.173116187 | 1.325474583 | 2.936696475 | 0.001987204 | 0.00712498 |
| AC093162.1 | 0.298231312 | 0.708336121 | 1.248002378 | 3.38E-07 | 7.64E-06 |
| TGM1 | 1.200663698 | 5.446840768 | 2.181587579 | 0.000445871 | 0.002181274 |
| TRAJ35 | 0.399814262 | 0.834203231 | 1.061068964 | 0.001388502 | 0.005386209 |
| GPR87 | 0.391077102 | 1.44683223 | 1.887372669 | 0.008273245 | 0.022071953 |
| CEL | 95.07631597 | 5.796270335 | -4.035889215 | 0.000974111 | 0.004044355 |
| AL139415.2 | 0.876023672 | 1.768993658 | 1.013887115 | 5.26E-06 | 6.48E-05 |
| SLC29A4 | 5.278831916 | 1.671430021 | -1.659135776 | 0.001450893 | 0.005585544 |
| F13A1 | 3.16612771 | 6.856932433 | 1.114843856 | 0.000145183 | 0.000877907 |
| TMPRSS11D | 0.57111179 | 1.219113776 | 1.093987701 | 0.000605797 | 0.002766194 |
| IGLV1-41 | 1.723432338 | 4.984395491 | 1.532133885 | 0.019415192 | 0.043815889 |
| A2ML1 | 2.798608457 | 5.721630951 | 1.031716787 | 0.00041566 | 0.002067135 |
| CR1 | 0.328518817 | 0.71742746 | 1.126856958 | 7.59E-07 | 1.42E-05 |
| HDAC11 | 4.770603437 | 2.300934487 | -1.051951858 | 9.95E-44 | 1.62E-39 |
| AC010422.3 | 6.420731815 | 3.130425612 | -1.036378921 | 0.000447516 | 0.002186688 |
| RGS13 | 0.320725872 | 0.821895891 | 1.357614924 | 5.29E-06 | 6.50E-05 |
| TRAJ1 | 0.347810896 | 0.734660006 | 1.078773607 | 0.000275307 | 0.001481622 |
| RNA5SP202 | 1.810916828 | 0.891347481 | -1.022660424 | 6.94E-06 | 8.04E-05 |
| SERPINB13 | 0.483534984 | 1.004094512 | 1.054202895 | 0.003682064 | 0.011598931 |
| DCLK1 | 0.392750464 | 0.876661667 | 1.158407185 | 0.000596927 | 0.00273801 |
| PROX1 | 4.822900169 | 1.913835918 | -1.333433802 | 0.002885206 | 0.00953245 |
| DPEP1 | 20.42089801 | 6.172353568 | -1.7261537 | 0.004889075 | 0.014543655 |
| LINC01781 | 0.529812743 | 1.097399646 | 1.050534566 | 0.000186344 | 0.001072883 |
| ADRA2C | 5.354449051 | 1.52294206 | -1.813877079 | 6.39E-05 | 0.000460274 |
| TEX45 | 0.761855363 | 0.379845405 | -1.004104759 | 1.17E-05 | 0.000121042 |
| SNORC | 4.665812262 | 1.466513897 | -1.669737516 | 1.42E-08 | 6.98E-07 |
| SERPINB2 | 1.94316928 | 5.286151032 | 1.443806058 | 0.007913947 | 0.021341245 |
| NLRP7 | 0.261836027 | 1.369829443 | 2.387260755 | 0.009636442 | 0.024946621 |
| SP5 | 9.230800942 | 3.789047846 | -1.284620476 | 0.008243224 | 0.02200632 |
| CITED1 | 1.130609241 | 0.45268348 | -1.320525832 | 0.000176682 | 0.001026349 |
| MTND4LP5 | 0.618665062 | 1.352823014 | 1.12874264 | 3.59E-06 | 4.74E-05 |
| FBXO2 | 10.35224176 | 4.645704773 | -1.155973833 | 5.52E-07 | 1.11E-05 |
| FCRL1 | 0.427884975 | 1.338096803 | 1.644887564 | 0.001455042 | 0.005598859 |
| CELP | 0.940701368 | 0.342492426 | -1.45766472 | 0.00786877 | 0.02123705 |
| GJC2 | 1.805668995 | 0.854750338 | -1.078958458 | 0.007001603 | 0.019376392 |
| ADH7 | 0.302585927 | 1.386245664 | 2.195766153 | 0.016062414 | 0.037678471 |
| FCRL2 | 0.296695007 | 0.660001791 | 1.153489292 | 0.003104537 | 0.0101131 |
| TRAJ34 | 0.287020658 | 0.661083499 | 1.203677927 | 0.000274419 | 0.001478802 |
| SNHG25 | 32.97586906 | 15.90701135 | -1.051747878 | 1.35E-07 | 3.73E-06 |
| CST1 | 117.7451672 | 57.43402642 | -1.035690236 | 0.000275162 | 0.001481335 |
| CLCA2 | 1.25368511 | 2.783268794 | 1.150605214 | 0.005696746 | 0.016437475 |
| KLK13 | 2.749160367 | 7.894715625 | 1.521896234 | 0.007798511 | 0.021089479 |
| TUBA3C | 0.63477358 | 1.786955291 | 1.493189551 | 0.001288924 | 0.005065256 |
| NOTUM | 85.56853818 | 17.05147795 | -2.327183653 | 2.89E-05 | 0.000246781 |
| CD22 | 1.218026396 | 3.218751969 | 1.401956011 | 0.001986117 | 0.007122656 |
| AC037198.2 | 0.427413939 | 0.892792808 | 1.062691447 | 1.24E-06 | 2.09E-05 |
| IL24 | 1.918616276 | 4.108910087 | 1.098689561 | 0.000426565 | 0.002105857 |
| TRAJ3 | 0.492654763 | 1.225109669 | 1.314261991 | 5.47E-06 | 6.69E-05 |
| SLCO4A1-AS1 | 6.945231552 | 2.79357521 | -1.313910132 | 0.001150382 | 0.004629463 |
| MAL | 7.903256479 | 20.12482419 | 1.348457047 | 0.000467703 | 0.002257485 |
| SYP | 1.578730892 | 0.647649565 | -1.285479966 | 0.000974111 | 0.004044355 |
| C10orf91 | 0.855107206 | 0.39473075 | -1.115236391 | 7.59E-07 | 1.42E-05 |
| CDX1 | 41.51161784 | 19.15981789 | -1.115431311 | 0.00405341 | 0.012506767 |
| CCNE1 | 18.49959028 | 6.584253777 | -1.490401472 | 0.005338581 | 0.01559238 |
| CXCL11 | 6.45232089 | 13.85283306 | 1.10229096 | 0.011427335 | 0.028629151 |
| HCN2 | 1.231802675 | 0.600248632 | -1.03713905 | 0.011363734 | 0.028491782 |
| CR2 | 3.146927776 | 7.758393768 | 1.301813932 | 0.009220737 | 0.024093225 |
| FCRL3 | 0.503025628 | 1.157087747 | 1.201794466 | 0.000200103 | 0.001135991 |
| GBP6 | 1.28798924 | 5.229986264 | 2.021686616 | 4.45E-06 | 5.64E-05 |
| BCYRN1 | 1.294745053 | 2.619322585 | 1.016525701 | 1.60E-05 | 0.000155642 |
| LINC01480 | 0.350076849 | 0.719763941 | 1.039852169 | 4.17E-07 | 8.98E-06 |
| SNORD93 | 0.442306439 | 0.975959549 | 1.141775108 | 1.08E-05 | 0.00011354 |
| B3GNT6 | 2.185043608 | 6.453532998 | 1.56242711 | 3.98E-05 | 0.000316076 |
| GCG | 0.238102573 | 3.251587875 | 3.771489295 | 0.00369843 | 0.01164371 |
| CLCA1 | 3.04045987 | 9.206687078 | 1.598392564 | 0.019380537 | 0.043761807 |
| FAM129C | 0.29352748 | 0.696558271 | 1.246748475 | 0.002714791 | 0.009098975 |
| FABP4 | 1.662512549 | 6.518032754 | 1.971071371 | 0.001840582 | 0.006680386 |
| STAP1 | 0.58650572 | 1.456649894 | 1.312437083 | 6.23E-05 | 0.000451189 |
| HUNK | 1.967355253 | 0.848023142 | -1.214081953 | 0.003611988 | 0.011424715 |
| SNORA14A | 1.054394847 | 0.487134976 | -1.11402175 | 7.15E-05 | 0.000501518 |
| C20orf204 | 1.235734281 | 0.358349354 | -1.785929897 | 0.01086613 | 0.027533179 |
| IGLL1 | 0.666223572 | 3.266188581 | 2.293529787 | 0.020911433 | 0.046380376 |
| RHOXF1-AS1 | 0.459742961 | 0.949664333 | 1.046590185 | 0.019722494 | 0.044342849 |
| FBXL16 | 3.547440518 | 1.695667527 | -1.064925169 | 0.000321308 | 0.001676418 |
| MS4A1 | 2.133226283 | 6.698036141 | 1.650701153 | 0.003084878 | 0.010067232 |
| LRMP | 1.057888576 | 2.443519835 | 1.207773134 | 7.55E-07 | 1.42E-05 |
| TLX1 | 1.944558063 | 0.852850298 | -1.189077883 | 0.014872012 | 0.035402494 |
| LINC00861 | 0.284952464 | 0.744316837 | 1.385195605 | 1.25E-07 | 3.55E-06 |
| FOXO6 | 2.674646093 | 1.272754181 | -1.071394203 | 3.76E-07 | 8.30E-06 |
| MYH7B | 1.171341495 | 0.420220432 | -1.478943527 | 0.001648835 | 0.006138063 |
| TMEM198 | 2.716639592 | 1.332325612 | -1.027876469 | 4.16E-08 | 1.60E-06 |
| GKN2 | 19.87028727 | 67.6893174 | 1.768315438 | 0.001896401 | 0.006853881 |
| AL034397.3 | 0.488156711 | 0.988355558 | 1.017685775 | 3.37E-06 | 4.52E-05 |
| IKZF3 | 2.88057493 | 6.119120524 | 1.086967529 | 0.000124369 | 0.00077812 |
| ASPSCR1 | 5.222708609 | 2.262286415 | -1.207016621 | 9.98E-12 | 1.88E-09 |
| MTCO1P42 | 0.545613284 | 2.551503865 | 2.225397152 | 0.000230312 | 0.001280195 |
| CD38 | 1.608416033 | 3.658497069 | 1.18561048 | 4.47E-06 | 5.66E-05 |
| SOSTDC1 | 1.520755308 | 3.742729778 | 1.299302851 | 0.000171235 | 0.001001564 |
| VGF | 5.961627715 | 2.008783506 | -1.569384199 | 0.002448439 | 0.008388007 |
| AC007991.2 | 2.476744986 | 5.179717042 | 1.064427957 | 0.000396556 | 0.001987306 |
| SCN7A | 0.320784092 | 0.677464759 | 1.078543303 | 0.001341928 | 0.005235743 |
| AXIN2 | 12.11091427 | 3.933212602 | -1.622527705 | 0.003797215 | 0.011883307 |
| GOLGA7B | 1.147604299 | 0.548496063 | -1.065072108 | 2.32E-06 | 3.40E-05 |
| INHBB | 9.176500409 | 2.897406863 | -1.663181779 | 0.015408334 | 0.03641236 |
| OGN | 7.683773374 | 15.79430921 | 1.039517966 | 0.006238964 | 0.017666188 |
| PARP15 | 0.739040475 | 1.682275459 | 1.186688671 | 2.48E-09 | 1.66E-07 |
| AC104958.2 | 4.033646418 | 1.833081481 | -1.13781371 | 0.000123874 | 0.000776221 |
| C8G | 7.157602404 | 2.291602953 | -1.643119304 | 0.004365365 | 0.013270122 |
| LINC01124 | 1.087586672 | 0.542083495 | -1.00454339 | 0.003947863 | 0.012253236 |
| AC125603.2 | 1.021189808 | 0.303667807 | -1.749685169 | 0.009609094 | 0.024903608 |
| PRR36 | 3.781445884 | 1.153607078 | -1.712786051 | 3.33E-08 | 1.34E-06 |
| GAL3ST2 | 3.334536844 | 1.501889334 | -1.150707878 | 0.002385697 | 0.00821828 |
| WNT11 | 7.28187484 | 3.164563229 | -1.20230355 | 0.012540647 | 0.0308427 |
| SNORA47 | 2.210071778 | 0.864755781 | -1.353728568 | 0.000553493 | 0.00257736 |
| LCN12 | 1.519940635 | 0.54709147 | -1.474161009 | 0.000914722 | 0.003851936 |
| TRAJ2 | 0.440359542 | 1.016395552 | 1.206708135 | 5.99E-06 | 7.17E-05 |
| MIR7848 | 0.436176369 | 0.920156746 | 1.076968031 | 1.26E-06 | 2.11E-05 |
| GLYATL2 | 0.515223321 | 1.140456172 | 1.146341203 | 0.018268236 | 0.041702605 |
| DLGAP1-AS5 | 0.787227851 | 1.653046161 | 1.070273844 | 0.004321513 | 0.01316779 |
| TP63 | 0.323125695 | 1.230494815 | 1.929071193 | 0.008484592 | 0.022506449 |
| BAMBI | 20.68567663 | 8.28506422 | -1.320047364 | 0.020243958 | 0.045270875 |
| H19 | 70.03531777 | 25.52796539 | -1.456004078 | 0.014439751 | 0.034540564 |
