# Supplementary material for: The histone acetylation-related gene signature predicts prognosis and immunotherapy response in stomach adenocarcinoma
Source: Front Oncol. 2025 Sep 2;15:1527253. doi: 10.3389/fonc.2025.1527253 (PMC12436397; doi:10.3389/fonc.2025.1527253)
Supplement: Supplementary file 2 [file Table2.docx]

| **Supplementary Table 2.** The list of histone acetylation‑related genes | | | |
| --- | --- | --- | --- |
| Gene symbol | Official full name | Ensembl ID | Gene type |
| ATF2 | activating transcription factor 2 | ENSG00000115966 | protein coding |
| BRD2 | bromodomain containing 2 | ENSG00000204256 | protein coding |
| BRD3 | bromodomain containing 3 | ENSG00000169925 | protein coding |
| BRD4 | bromodomain containing 4 | ENSG00000141867 | protein coding |
| BRDT | bromodomain testis associated | ENSG00000137948 | protein coding |
| CIITA | class II major histocompatibility complex transactivator | ENSG00000179583 | protein coding |
| CLOCK | clock circadian regulator | ENSG00000134852 | protein coding |
| CREBBP | CREB binding protein | ENSG00000005339 | protein coding |
| EP300 | E1A binding protein p300 | ENSG00000100393 | protein coding |
| HAT1 | histone acetyltransferase 1 | ENSG00000128708 | protein coding |
| HDAC1 | histone deacetylase 1 | ENSG00000116478 | protein coding |
| HDAC2 | histone deacetylase 2 | ENSG00000196591 | protein coding |
| HDAC3 | histone deacetylase 3 | ENSG00000171720 | protein coding |
| HDAC4 | histone deacetylase 4 | ENSG00000068024 | protein coding |
| HDAC5 | histone deacetylase 5 | ENSG00000108840 | protein coding |
| HDAC6 | histone deacetylase 6 | ENSG00000094631 | protein coding |
| HDAC7 | histone deacetylase 7 | ENSG00000061273 | protein coding |
| HDAC8 | histone deacetylase 8 | ENSG00000147099 | protein coding |
| HDAC9 | histone deacetylase 9 | ENSG00000048052 | protein coding |
| HDAC10 | histone deacetylase 10 | ENSG00000100429 | protein coding |
| HDAC11 | histone deacetylase 11 | ENSG00000163517 | protein coding |
| KAT2A | lysine acetyltransferase 2A | ENSG00000108773 | protein coding |
| KAT2B | lysine acetyltransferase 2B | ENSG00000114166 | protein coding |
| KAT5 | lysine acetyltransferase 5 | ENSG00000172977 | protein coding |
| KAT6A | lysine acetyltransferase 6A | ENSG00000083168 | protein coding |
| KAT6B | lysine acetyltransferase 6B | ENSG00000156650 | protein coding |
| KAT7 | lysine acetyltransferase 7 | ENSG00000136504 | protein coding |
| KAT8 | lysine acetyltransferase 8 | ENSG00000103510 | protein coding |
| NCOA1 | nuclear receptor coactivator 1 | ENSG00000084676 | protein coding |
| NCOA2 | nuclear receptor coactivator 2 | ENSG00000140396 | protein coding |
| NCOA3 | nuclear receptor coactivator 3 | ENSG00000124151 | protein coding |
| OGA | O-GlcNAcase | ENSG00000198408 | protein coding |
| SIRT1 | sirtuin 1 | ENSG00000096717 | protein coding |
| SIRT2 | sirtuin 2 | ENSG00000068903 | protein coding |
| SIRT3 | sirtuin 3 | ENSG00000142082 | protein coding |
| SIRT4 | sirtuin 4 | ENSG00000089163 | protein coding |
| SIRT5 | sirtuin 5 | ENSG00000124523 | protein coding |
| SIRT6 | sirtuin 6 | ENSG00000077463 | protein coding |
| SIRT7 | sirtuin 7 | ENSG00000187531 | protein coding |
| TAF1 | TATA-box binding protein associated factor 1 | ENSG00000147133 | protein coding |
