# Supplementary material for: The histone acetylation-related gene signature predicts prognosis and immunotherapy response in stomach adenocarcinoma
Source: Front Oncol. 2025 Sep 2;15:1527253. doi: 10.3389/fonc.2025.1527253 (PMC12436397; doi:10.3389/fonc.2025.1527253)
Supplement: Supplementary file 3 [file Table3.docx]

| **Supplementary Table 3.** Eight-Gene Prognostic Model | | | |
| --- | --- | --- | --- |
| Gene | Official full name | Ensembl ID | Coef |
| ASCL2 | achaete-scute family bHLH transcription factor 2 | ENSG00000183734 | -0.015955372 |
| GPR87 | G protein-coupled receptor 87 | ENSG00000138271 | 0.107651162 |
| F13A1 | coagulation factor XIII A chain | ENSG00000124491 | 0.051803639 |
| HDAC11 | histone deacetylase 11 | ENSG00000163517 | -0.084605503 |
| DCLK1 | doublecortin like kinase 1 | ENSG00000133083 | 0.111253472 |
| GCG | glucagon | ENSG00000115263 | 0.088183777 |
| FABP4 | fatty acid binding protein 4 | ENSG00000170323 | 0.024354792 |
| AXIN2 | axin 2 | ENSG00000168646 | -0.087962004 |
