# Supplementary material for: The histone acetylation-related gene signature predicts prognosis and immunotherapy response in stomach adenocarcinoma
Source: Front Oncol. 2025 Sep 2;15:1527253. doi: 10.3389/fonc.2025.1527253 (PMC12436397; doi:10.3389/fonc.2025.1527253)
Supplement: Supplementary file 4 [file Table4.docx]

| **Supplemental Table 4**. The primers and siRNAs used in this study. | |
| --- | --- |
| Primer/siRNA | Sequence (5’-3’) |
| DCLK1 forward | ACTTCGACGAGCGGGATAAG |
| DCLK1 reverse | GGGCCTCAAAAGATCGGAACC |
| β-Actin forward | CACCATTGGCAATGAGCGGTTC |
| β-Actin reverse | AGGTCTTTGCGGATGTCCACGT |
| Si-DCLK1-1 sense | UCUGUCGGAUAACGUGAAUUU |
| Si-DCLK1-1 antisense | AAAUUCACGUUAUCCGACAGA |
| Si-DCLK1-2 sense | ACCCGAACUCUGUCGGAUAAC |
| Si-DCLK1-2 antisense | GUUAUCCGACAGAGUUCGGGU |
