# Supplementary material for: The histone acetylation-related gene signature predicts prognosis and immunotherapy response in stomach adenocarcinoma
Source: Front Oncol. 2025 Sep 2;15:1527253. doi: 10.3389/fonc.2025.1527253 (PMC12436397; doi:10.3389/fonc.2025.1527253)
Supplement: Supplementary file 5 [file Table5.docx]

| **Supplementary Table 5**. Clinicopathological characteristics of DCLK1 in gastric cancer | | | | |
| --- | --- | --- | --- | --- |
| Clinicopathological parameters | Cases | DCLK1 expression | | p Value |
|  |  | High(15) | Low(13) |  |
|  | 28 | No. of patients | No. of patients |  |
| **Age** |  |  |  | >0.9999 |
| ≤ 60 | 13 | 7 | 6 |  |
| > 60 | 15 | 8 | 7 |  |
| **Sex** |  |  |  | >0.9999 |
| Male | 16 | 9 | 7 |  |
| Female | 12 | 6 | 6 |  |
| **Differentiation** |  |  |  | 0.1513 |
| Well/Moderate | 15 | 6 | 9 |  |
| Poor | 13 | 9 | 4 |  |
| **Borrmann’s Type** |  |  |  | 0.0011* |
| 0-II | 10 | 1 | 9 |  |
| III-IV | 18 | 14 | 4 |  |
| **Depth of tumor invasion** |  |  |  | 0.0418* |
| pT1–2 | 9 | 2 | 7 |  |
| pT3–4 | 19 | 13 | 6 |  |
| **Lymph node metastasis** |  |  |  | 0.0011* |
| Negative | 10 | 1 | 9 |  |
| Positive | 18 | 14 | 4 |  |
| **TNM Stage** |  |  |  | 0.0004* |
| I-II | 13 | 2 | 11 |  |
| III | 15 | 13 | 2 |  |
|  |  |  |  |  |
|  |  |  |  |  |
|  |  |  |  |  |
